# Resistance analysis of cherry rootstock ‘CDR-1’ (*Prunus mahaleb*) to crown gall disease

**DOI:** 10.1186/s12870-020-02673-0

**Published:** 2020-11-12

**Authors:** Chenglin Liang, Tian Wan, Rendun Wu, Mei Zhao, Yue Zhao, Yuliang Cai

**Affiliations:** College of Horticulture, Northwest A&F University, Yangling, 712100 Shaanxi Province China

**Keywords:** Crown gall disease, *A. tumefaciens*, Gene function, Lignin biosynthetic pathway, *Pm4CL2*, Resistance

## Abstract

**Background:**

Crown gall disease, caused by the pathogenic bacterium *Agrobacterium tumefaciens*, is responsible for extensive economic losses in orchards. Cherry rootstock ‘CDR-1’ (*Prunus mahaleb*) shows high resistance but the mechanism remains unclear. Here, we examined the morphology of pathogen-infected root neck surface, determined the activity of 10 defense-related enzymes and the content of salicylic acid (SA) and jasmonic acid (JA), and also applied transcriptome analysis, transient expression and transgenic verification to explore the crown gall resistance genes in ‘CDR-1’ plants.

**Results:**

In our study, peroxidase increased in the first 10 days, while phenylalanine ammonialyase and lipoxygenase increased in the first 15 days post-infection. Four key enzymes in the AsA-GSH cycle also responded, to a certain extent; although JA content increased significantly after the treatment, the SA content did not. In a follow-up transcriptome analysis, the differentially expressed genes *Pm4CL2*, *PmCYP450*, *PmHCT1*, *PmHCT2*, and *PmCAD* were up-regulated. Based on the above results, we focused on the lignin biosynthetic pathway, and further measured lignin content, and found it increased significantly. The *Pm4CL2* gene was used to conduct transient expression and transgenic experiments to verify its function in crown gall disease resistance. It showed the relative expression of the treatment group was almost 14-fold that of the control group at 12 h post-treatment. After the infection treatment, clear signs of resistance were found in the transgenic lines; this indicated that under the higher expression level and earlier activation of *Pm4CL2*, plant resistance was enhanced.

**Conclusions:**

The crown gall resistance of ‘CDR-1’ is likely related to the lignin biosynthetic pathway, in which *Pm4CL2* functions crucially during the plant defense response to the pathogen *A. tumefaciens*. The results thus offer novel insights into the defense responses and resistance mechanism of cherry rootstock ‘CDR-1’ against crown gall disease.

**Supplementary information:**

**Supplementary information** accompanies this paper at 10.1186/s12870-020-02673-0.

## Background

Crown gall disease was identified long ago as a bacterial plant disease [1], and its pathogenic bacterium is *Agrobacterium tumefaciens*, which mainly infects dicots. This disease often results in severe economic losses to the production of cherry and other fruit trees [2–4]. Crown gall disease starts with the attachment of *A. tumefaciens* to plant cell. And then the transfer DNA, a portion of the Ti plasmid, will be integrated into the plant genome. Finally, the symptomatic tumors form and grow [5].

Crown gall disease affects many fruit trees and causes extensive economic losses in nurseries. In a previous study, 11 tree species were surveyed. The highest disease incidence was found in peach (*Prunus persica* [L.] Batsch), almond (*P. dulcis* D Webb), cherry (*P. avium* L.), apple (*Malus sylvestris* Mill) and olive (*Olea europaea* L.) [6]. It was also found the rootstock of peach, cherry, apple and pear (*Pyrus communis* L.) trees was a influence factor contributing to the significant differences in the frequency of galled plants.

Plants are often exposed to many various bacterial, viral, and fungal pathogens but have evolved potent defense systems to protect themselves [7]. In defense responses of plants, the identification of microbial pathogens plays a key role, as it “turns on” the signal transduction pathway which activates the expression of numerous pathogen-responsive genes [8, 9]. These disease resistance genes are crucial for identifying the effector proteins during the process of pathogen infection [7].

Many biotechnological strategies have been developed and applied in the attempt to control crown gall disease. In transformation experiments, the truncated genes involved in T-DNA transfer have been used to induce plant resistance to crown gall disease [10, 11], and inactivating the oncogenes could prevent tumor formation [12]. Therefore, to obtain plants that are resistant to crown gall disease, much research has been devoted to producing sense and antisense strands of the oncogene sequence by placing these sequences between opposing strong constitutive promoters [13], or to silencing the involved bacterial oncogenes by using premature stop codons [14]. The study of Niemeyer et al. (2014) demonstrated a successful reprogramming of the viral *N* gene response against crown gall disease [9]. In recent years, Rosalia Deeken’s group has been working on the molecular mechanism between crown gall disease and *A. tumefaciens* in *Arabidopsis thaliana* [8, 15–18]. Pathogen infection always induces response of plant hormones. Lee et al. (2009) explored the physiological changes and adaptations on the aspect of SA, JA, ethylene (ET), and auxin (indole-3-acetic acid, IAA) with changes in the *Arabidopsis thaliana* transcriptome during tumor development [5].

At present, planting resistant cultivars and developing biological antagonists both are effective measures to control crown gall disease in orchards [3]. The existing biological antagonists are mainly used for prevention but they act poorly as a treatment. So the crown gall-resistant cultivars in agriculture were in need [19]. Previous studies have reported crown gall-resistant cultivars for apple, peach, plum, grapevine, aspen, and roses [20–27]. Crown gall resistance has been assessed in accessions of 20 *Prunus* species [21]. And it was found that when the strains K12 and C58 of *A. tumefaciens* were used to infect the main stems or lateral branches of seedlings, the incidence of resistance was up to 30% in some accessions of *P. mahaleb*. The cherry breeding resource plant *P. mahaleb* is a cosmopolitan cherry rootstock. In northwest China, it has become one of the main sweet cherry rootstocks because of its excellent biological traits, such as strong resistance to crown gall disease, dwarfing ability and salinity among other desirable traits [28]. By systematic classification of cherry species, *P. mahaleb* belongs to the III. *Cerasus* subgenus, Section 5 Mahaleb Focke [29]. It is a deciduous tree or large shrub, growing to 2–10 m (rarely up to 12 m) tall with a trunk up to 40 cm diameter. In most cherry growing countries, mahaleb cherry is used to be rootstock of sweet and sour cherries [28]. This rootstock showed strong resistance to crown gall disease in cherry production, but little is known about its mechanism of crown gall resistance. Furthermore, the actual genes (without modification) underpinning resistance to crown gall have not yet been reported.

In this study, we focused on cherry rootstock ‘CDR-1’ (*P. mahaleb*), the natural hybrid cultivar of *P. mahaleb*. The objective of our study was to investigate the resistance mechanism of ‘CDR-1’ to crown gall disease. Here, we carried out morphological observations, physiological and biochemical analyses, gene expression analysis and transcriptomic analysis in ‘CDR-1’, and conducted transient expression and transgenic verification in tobacco. Our results provide evidence that the crown gall resistance of ‘CDR-1’ is likely related to the lignin biosynthetic pathway.

## Results

### Morphological observations

**Fig. 1:**
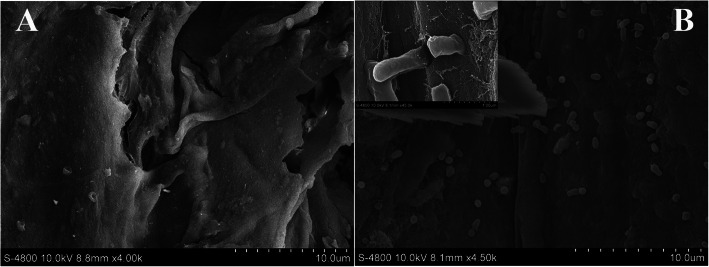
Morphological observation of the wound surface viewed under a field emission scanning electron microscope. **a** Images of the wound in wounded control group at 5 days post-infection (dpi) at a magnification of × 4.00 k. **b** Images of the wound in treatment with inoculation at 5 dpi at a magnification of × 4.50 k

Morphological observation using field emission scanning electron microscopy (FESEM) revealed *A. tumefaciens* cells were entirely absent from the wounded control group at 5 days post-infection (dpi) at a magnification of × 4.00 k (Fig. 1a), but many cells attached to the wound surface in treatment with inoculation at a magnification of × 4.50 k (Fig. 1b). Notably, the vertical invasive mode of *A. tumefaciens* entering into a given ‘CDR-1’ plant via the wound site was captured by FESEM (Fig. 1b).

### Biochemical analysis

**Fig. 2:**
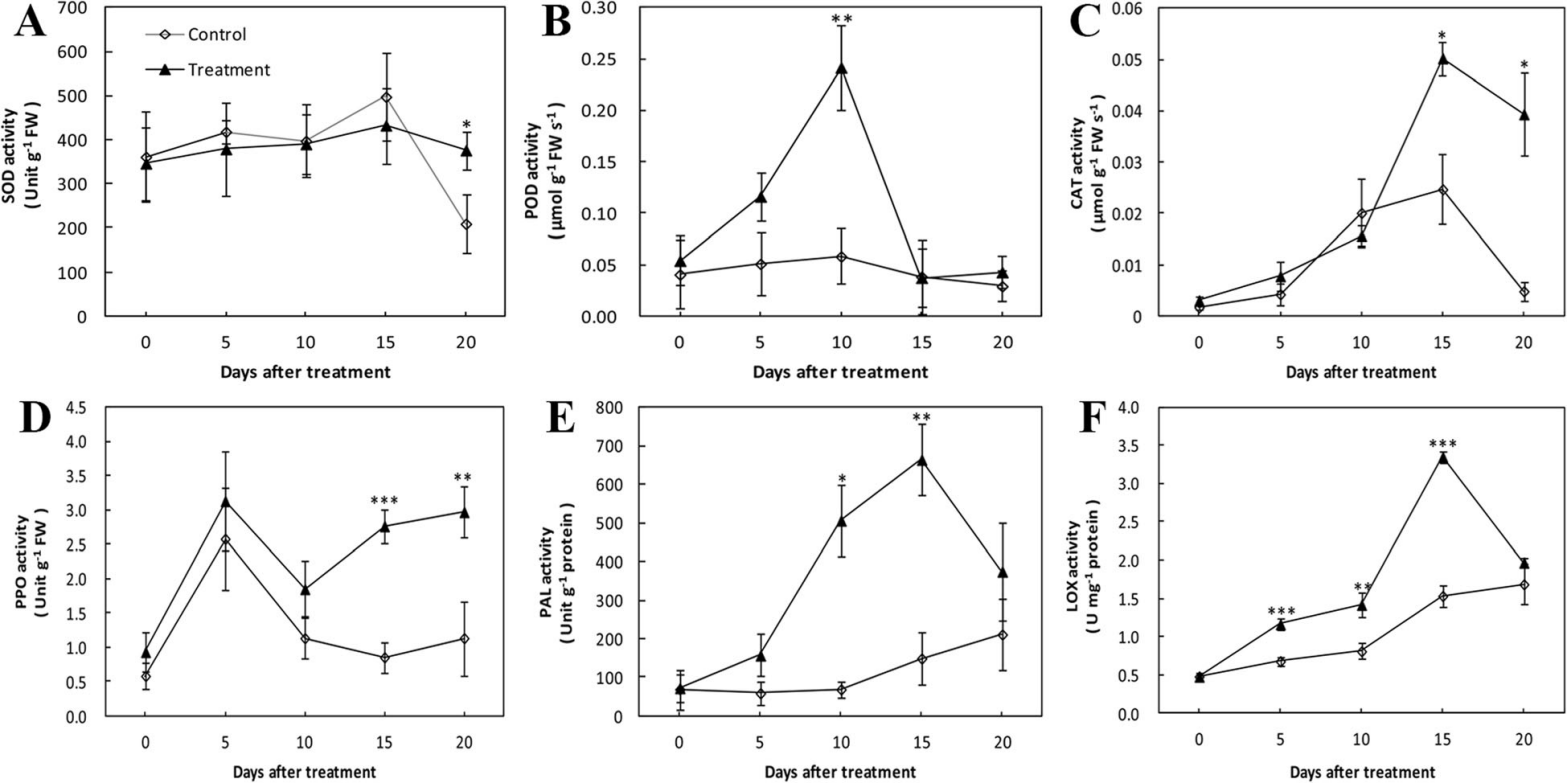
Effect of *Agrobacterium tumefaciens* infection on the activity of defense-related enzymes in cherry rootstock ‘CDR-1’ (*Prunus mahaleb*) at 0, 5, 10, 15, and 20 dpi. **a** SOD; **b** POD; **c** CAT; **d** PPO; **e** PAL; **f** LOX. Data symbols are the mean ± standard deviation (SD) (*n* = 3). Significant differences are indicated with asterisks (* *P* < 0.05, ** *P* < 0.01, *** *P* < 0.001)

The treatment with inoculation and wounded control groups had similar superoxide dismutase (SOD) activity. There was no significant difference between them except at 20 dpi when SOD activity in treatment group was higher than that in wounded control group (Fig. 2a). However, peroxidase (POD) activity in treatment group sharply increased to a peak at 10 dpi, but then decreased rapidly and sustained a relatively stable level, while in the wounded control it remained at a low and stable level during the whole period (Fig. 2b). Catalase (CAT) activity in both the wounded control and treatment group sharply increased in the first 15 days, and then decreased during the remainder of the hours. But a significantly higher CAT activity was observed in treatment group compared to the control at 15 and 20 dpi (Fig. 2c). In the treatment group, polyphenol oxidase (PPO) activity increased in the first 5 days and declined until 10 dpi, but then gradually increased until the end of the experiment. The wounded control group followed a similar trend in the first 10 days, and there was no significant difference compared to the treatment with inoculation. From 10 to 20 dpi, PPO activity in the wounded control remained at a low and stable level (Fig. 2d). The activity of phenylalanine ammonialyase (PAL) in the treatment gradually increased in the first 5 days and sharply increased to a peak until 15 dpi, and then rapidly decreased in the final days. PAL activity in the wounded control remained at a low level but a slightly increase was observed from 10 to 20 dpi. Thus, a relatively higher level of PAL activity was observed during the whole period (Fig. 2e). Lipoxygenase (LOX) activity in both the wounded control and treatment group gradually increased in the first 10 days. LOX activity in the treatment with inoculation sharply increased to a peak at 15 dpi, and then rapidly declined over the later days, while that in the wounded control showed a gradual increase in the next 10 days. In an overall view, LOX activity in the treatment was significantly higher than that in the wounded control during the whole period except at 20 dpi (Fig. 2f).

**Fig. 3:**
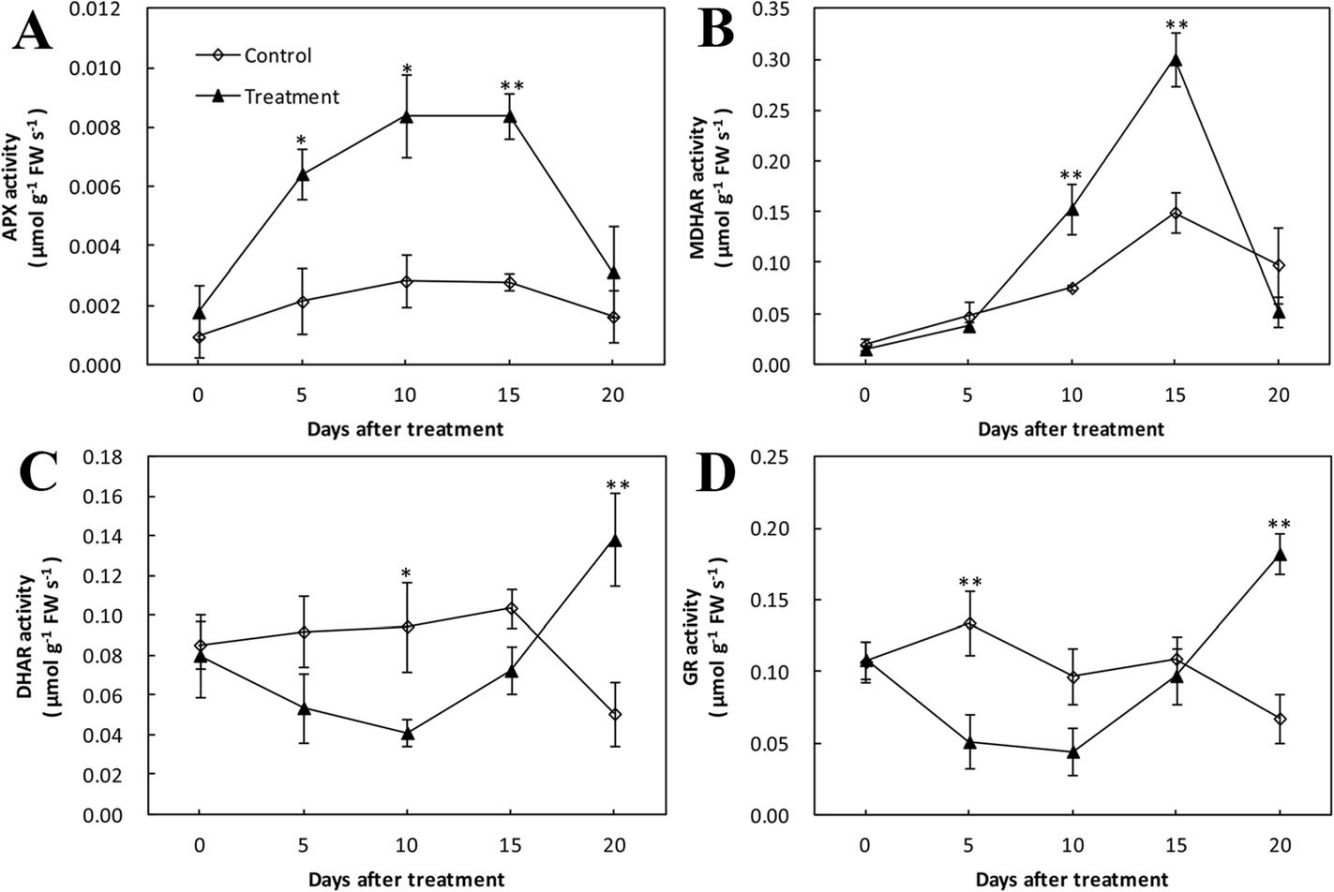
Effect of *Agrobacterium tumefaciens* infection on the activity of key enzymes in the AsA–GSH cycle of cherry rootstock ‘CDR-1’ (*Prunus mahaleb*) at 0, 5, 10, 15, and 20 dpi. **a** APX; **b** MDHAR; **c** DHAR; **d** GR. Data symbols represent the mean ± SD (*n* = 3). Significant differences are indicated with asterisks (* *P* < 0.05, ** *P* < 0.01)

The ascorbate peroxidase (APX) activity in the treatment with inoculation sharply increased in the first 5 days and gradually increased from 5 to 15 dpi, and then rapidly decreased until the end of the experiment, while in the wounded control it remained at a low and relatively stable level during the whole period (Fig. 3a). The monodehydroascorbate reductase (MDHAR) activity in treatment group was steady for the first 5 dpi, and sharply increased to a peak at 15 dpi, and then rapidly decreased over the later days. The infection treatment significantly increased MDHAR activity of ‘CDR-1’ from 10 to 15 dpi in contrast to the wounded control (Fig. 3b).

The activity of dehydroascorbate reductase (DHAR) and glutathione reductase (GR) both declined by almost 50% by day 10 in treatment group before rising. DHAR and GR activity in the treatment with inoculation both were significantly higher compared to the wounded control at 20 dpi. In the wounded control, DHAR activity remained relatively steady for the first 15 days and then declined, while GR activity slightly fluctuated for the first 15 days and then likewise declined (Fig. 3c and d).

**Fig. 4:**
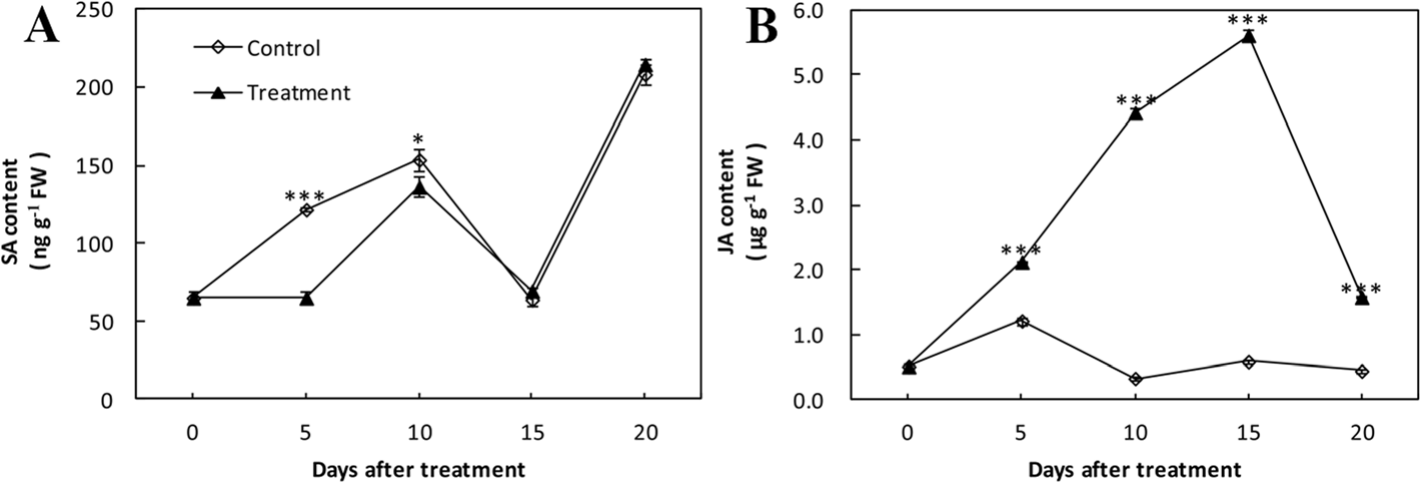
Effect of *Agrobacterium tumefaciens* infection on the content of phytohormones in cherry rootstock ‘CDR-1’ (*Prunus mahaleb*) at 0, 5, 10, 15, and 20 dpi. **a** SA; **b** JA. Data symbols represent the mean ± SD (*n* = 3). Significant differences are indicated with asterisks (* *P* < 0.05, *** *P* < 0.001)

The total SA content both in wounded control and treatment group showed a fluctuation during the whole period, with peaks at 10 and 20 dpi. SA content in the treatment remained unchanged for the first 5 dpi, increased at 10 dpi, decreased from 10 to 15 dpi, and then sharply increased over the later days. In the wounded control, it gradually increased for the first 10 dpi which was significantly higher in contrast to the treatment with inoculation, and followed a similar trend with treatment group for the next 10 days (Fig. 4a). The JA content in treatment group sharply increased to a peak for the first 15 dpi, and then rapidly decreased over the later days, while in the wounded control it remained at a low level with a slight fluctuation. Thus, a significantly higher level of JA content was observed in treatment group during the whole period (Fig. 4b).

### Transcriptomic analysis

**Fig. 5:**
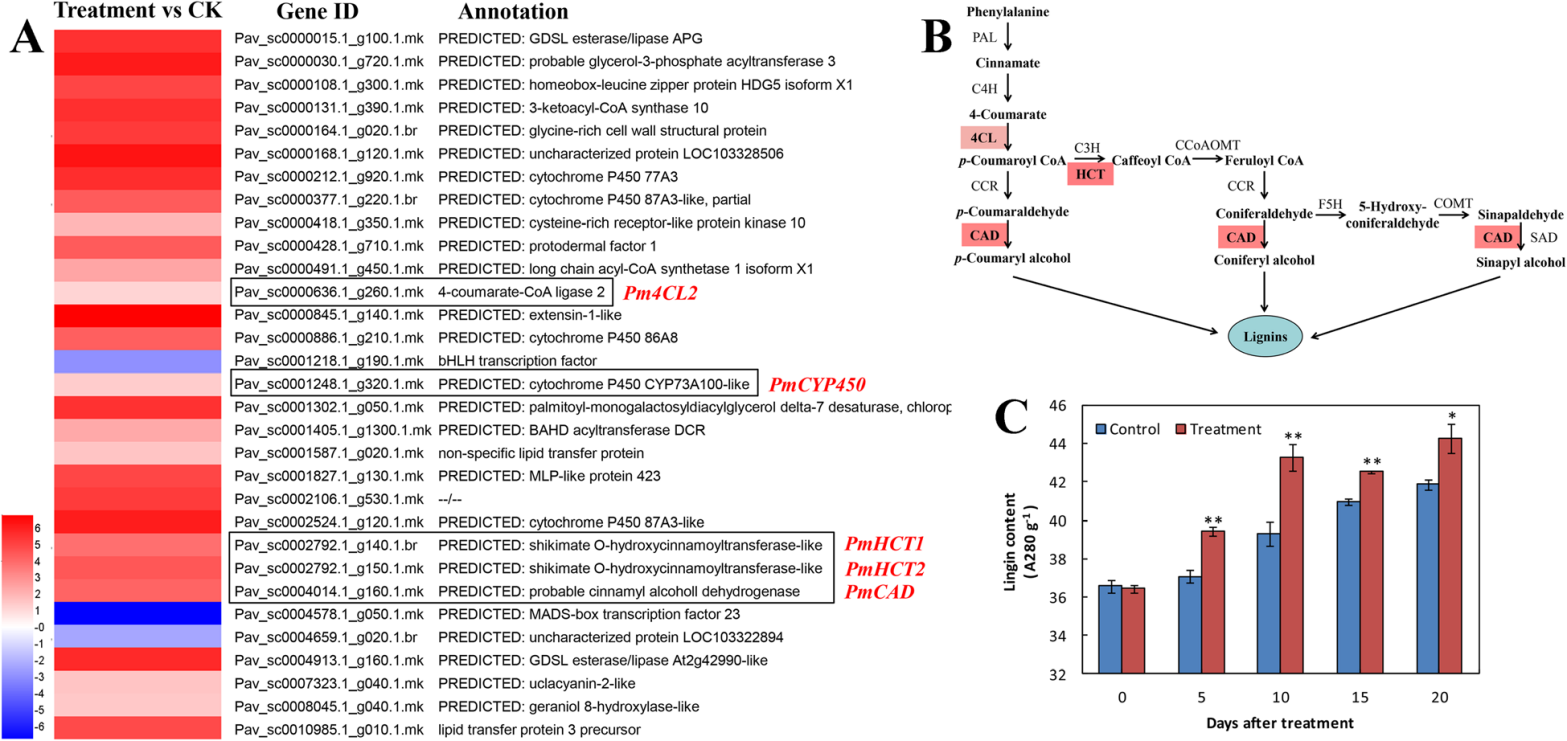
Functional annotation of differentially expressed genes (DEGs) and the lignin biosynthetic simplified pathway. **a** Expression profiles and annotations of DEGs. The log_2_ [fold change (FC)] value is represented by color depths, with red for upregulation and green for downregulation. **b** Lignin biosynthetic pathway. The enzymes marked in red correspond to five DEGs in this pathway. **c** Lignin content of cherry rootstock ‘CDR-1’ (*Prunus mahaleb*) at 0 dpi, 5 dpi, 10 dpi, 15 dpi and 20 dpi after the *Agrobacterium tumefaciens* infection treatment. Bars are the mean ± SD (*n* = 3). Significant treatment effects are indicated with asterisks (* *P* < 0.05, ** *P* < 0.01)

To obtain a general overview of the ‘CDR-1’ plant transcriptome in response to pathogen infection, the infected root neck tissues were harvested at 5 dpi. From these samples, we obtained 43,331,742 to 63,922,658 total reads, of which more than 63% were mapped to the reference cherry (*P. avium*) genome (Additional file 1: Table S1). Further, 40 differentially expressed genes (DEGs) were identified with an absolute value of log_2_ (fold change) ≥ 1 and a false discovery rate of < 0.001. By GO annotation, KEGG pathway, and enrichment analysis, the predicted functions of DEGs were obtained. These DEGs were distributed into 30 functional terms according to the GO annotation. Among them, 16 terms were under biological process, 13 terms were under molecular function, and 1 term was under cellular component (Additional file 1: Fig. S1). The genes in the biological process group were mainly involved in metabolic and oxidation-reduction processes. The molecular function terms were related to transition metal ion binding: in particular, iron ion binding, transferase activity, heme binding, oxidoreductase activity and tetrapyrrole binding were all significantly enriched GO terms. According to the KEGG pathway and enrichment analysis, DEGs were significantly enriched in the pathways of fatty acid metabolism and phenylpropanoid biosynthesis (Additional file 1: Fig. S2). Among the 40 DEGs there were 37 upregulated genes and 3 downregulated genes, all of which we annotated successfully (Fig. 5a).

Given the KEGG pathway and enrichment analysis results, combined with those of POD, PPO, PAL enzyme activity, we then focused on the pathway of phenylpropanoid biosynthesis (Fig. 5b, Additional file 1: Fig. S3). Specifically, we measured the relative expression of the genes—*PmPAL1*, *PmPAL2*, *Pm4CL1*, *Pm4CL2*, *PmCAD1* and *PmCAD2*—encoding the key enzymes PAL, 4CL (4-coumarate: CoA ligase), CAD (cinnamyl alcohol dehydrogenase) in this pathway. Fig. S4 shows their expression levels of these genes in ‘CDR-1’ plants. The mRNA levels of *PmPAL1*, *PmPAL2*, *Pm4CL1*, *Pm4CL2*, *PmCAD1*, and *PmCAD2* were all significantly upregulated in the treatment group at 5 dpi, with the strongest response occurring for *Pm4CL2*, whose expression was 12-fold that of the wounded control group (Additional file 1: Fig. S4). In this study, the genes *Pm4CL2* (ID: Pav_sc0000636.1_g260.1.mk, LOC110758567), *PmCYP450* (ID: Pav_sc0001248.1_g320.1.mk, LOC110766184), *PmHCT1* (Pav_sc0002792.1_g140.1.br, LOC110774309), *PmHCT2* (Pav_sc0002792.1_g150.1.mk, LOC103335795), and *PmCAD* (Pav_sc0004014.1_g160.1.mk, LOC110744673) screened from *P. mahaleb* were the DEGs in the phenylpropanoid biosynthesis pathway.

Next, we measured the lignin content of plants infected with *A. tumefaciens*. This generally became elevated over time, showing significant effects of the treatment compared with the control as early as day 5 post infection (Fig. 5c). The gene *Pm4CL2*, catalysing the biosynthesis of lignin monomers, was thus chosen as the target gene for use in the overexpression experiment below.

### Hypothesis verification through tobacco genetic transformation

**Fig. 6:**
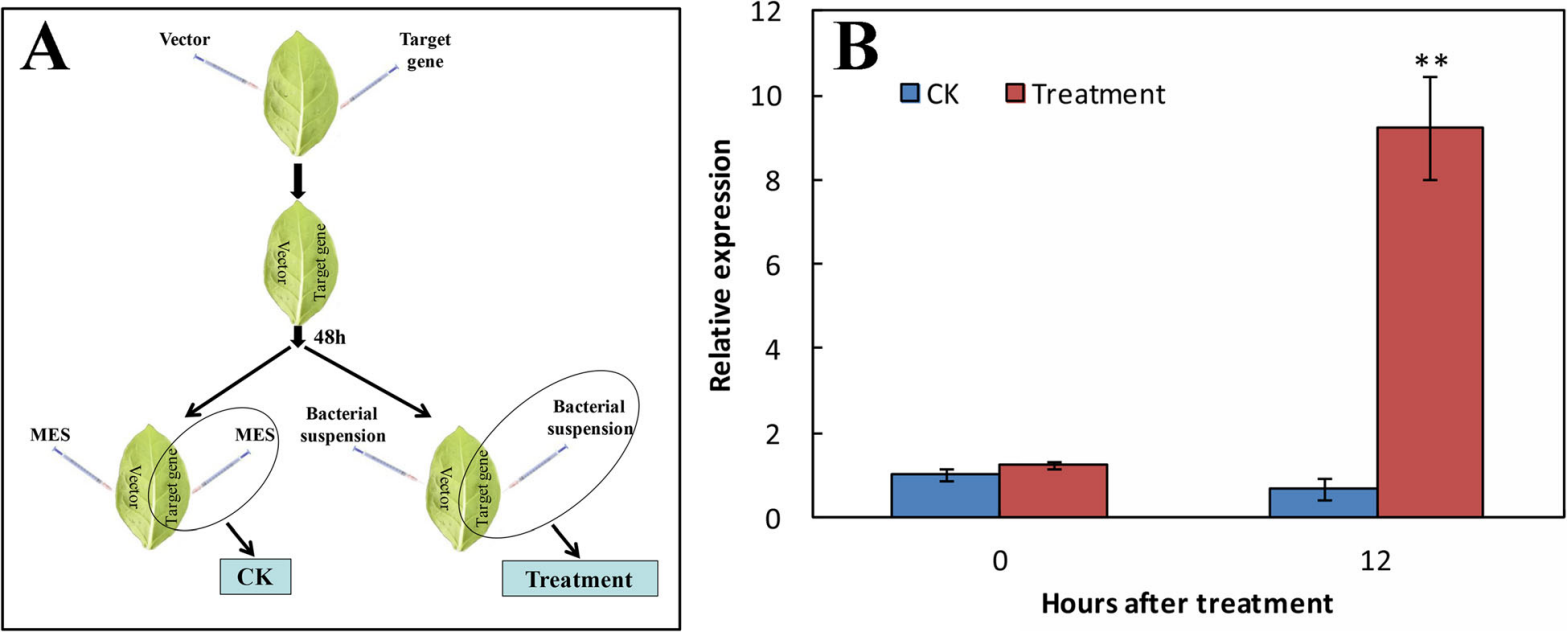
Operation procedure of transient expression in tobacco leaves (**a**) and the relative expression levels of the target gene *Pm4CL2* in the control (CK) and treatment groups at 12 h after the *Agrobacterium tumefaciens* infection treatment (**b**). Data represent mean ± SD (*n* = 3). Significant treatment effects are indicated with asterisks (** *P* < 0.01)

The transient expression analysis revealed the relative expression of treatment group was approximately 14-fold that of the control group at 12 h post-treatment (Fig. 6).

To investigate the functioning of *Pm4CL2* under the stress of *A. tumefaciens*, we transformed the target gene *Pm4CL2* into tobacco (*Nicotiana tabacum* L., cv SR1) and generated *Pm4CL2*-overexpressing transgenic tobacco lines (Additional file 1: Fig. S5). Among them, two independent lines (4CL2–1 and 4CL2–2) were chosen to conduct the experiments that follow. The amplified fragment length of *Pm4CL2* was 1815 bp as determined by PCR (Additional file 1: Fig. S6).

**Fig. 7:**
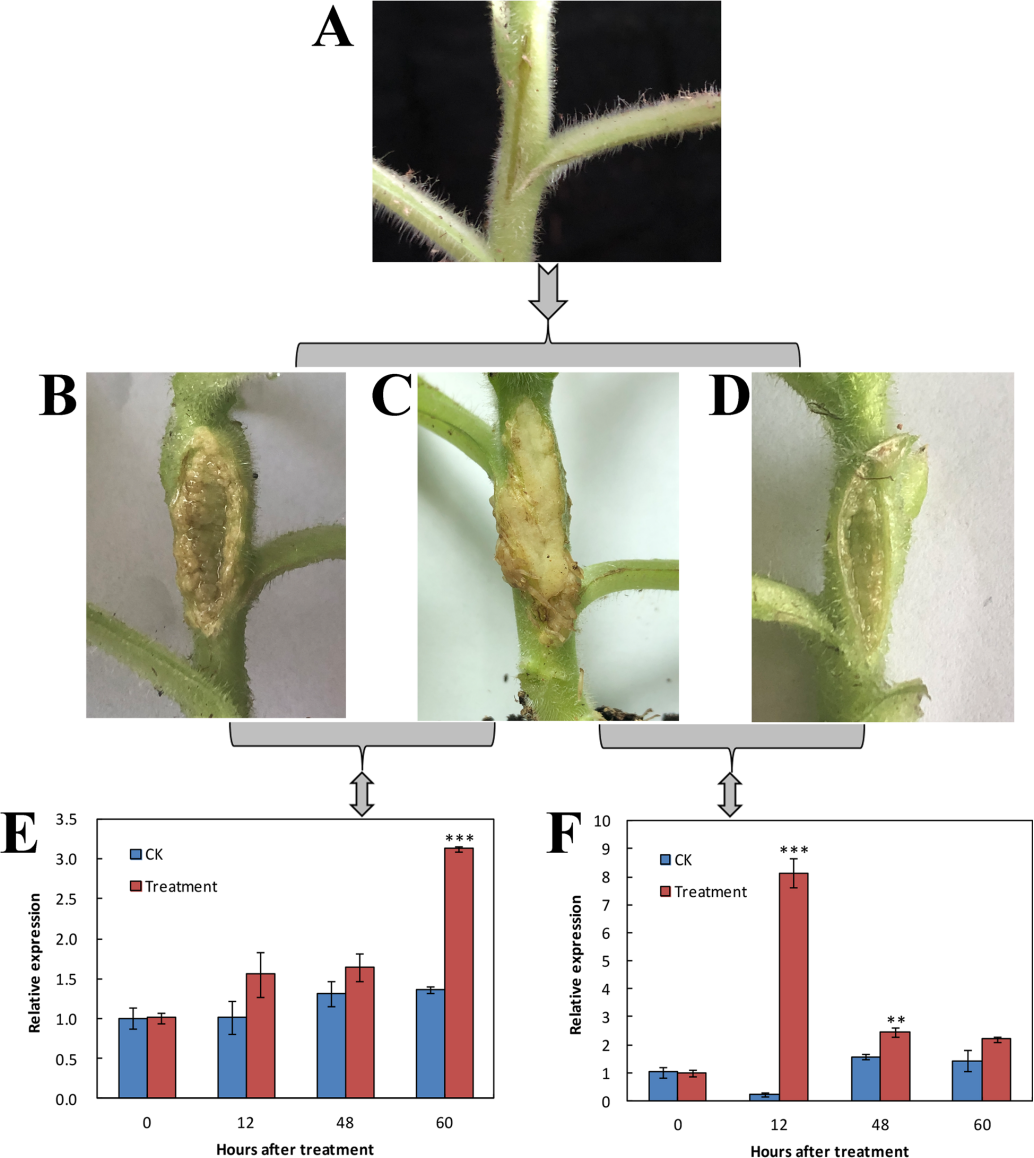
Resistance verification of transgenic tobacco with the target gene *Pm4CL2* to crown gall disease. **a** The stems of tobacco plants infected with the oncogenic *Agrobacterium tumefaciens*. **b** Symptom of transgenic line 4CL2–1 at 14 dpi. **c** Symptom of wild-type (WT) tobacco plants at 14 dpi. **d** Symptom of transgenic line 4CL2–2 at 14 dpi. **e** Relative expression levels of transgenic line 4CL2–1 and WT tobacco plants at 0 h, 12 h, 48 h, 60 h after the infection treatment. **f** Relative expression of transgenic line 4CL2–2 and WT tobacco plants at 0 h, 12 h, 48 h, and 60 h after the infection treatment. Bars are the mean ± SD (*n* = 3). Significant treatment effects are indicated with asterisks (** *P* < 0.01, *** *P* < 0.001)

To test the susceptibility to *Agrobacterium*-induced crown gall disease on the plant, the stems of transgenic plants and wild-type (WT) tobacco plants were infected with the oncogenic *A. tumefaciens* (Fig. 7a). Two weeks after infection, in the transgenic line 4CL2–1, several galls were visible on the wound site with the damage degree III (Fig. 7b), while the young galls of the WT plant grew rapidly and had filled the whole wound with the damage degree V (Fig. 7c). However, the symptoms of transgenic line 4CL2–2 were less obvious with just a few small galls that appeared sporadically on the wound, and the damage degree was only I (Fig. 7d). After 0 h, 12 h, 48 h, and 60 h, the relative expression of both transgenic lines was significantly higher than that of counterpart WT plants. After infection, the relative expression of transgenic line 4CL2–1 increased gradually, reaching a peak at 60 h (Fig. 7e), whereas that of transgenic line 4CL2–2 peaked much earlier, at 12 h (Fig. 7f).

## Discussion

Plants can be attacked by many organisms over their lifetimes. Pathogenic bacteria could invade plant tissues and proliferate in the extracellular space. Plants have evolved the immune system to recognize and limit the growth of pathogens [30]. During the first several hours after pathogen infection of plants, to a certain extent, their defense response pathways become activated, but this depends on the plant system [8].

In a preliminary experiment, ‘CDR-1’ resisted crown gall disease more strongly than did ‘Gisela 6’, prompting us to use the former plant as material for our resistance analysis. In this study, crown gall development was accompanied by profound changes in the morphology, defense-related enzymes, and phytohormones of infected seedlings, and their defense responses strengthened over time. After inoculation, we found some bacteria still attached to the wound surface in the treatment group at 5 dpi which tried to invade the plant. It suggested that *A. tumefaciens* infection was chronic compared to the single pulse of stress induced by mechanical damage.

In this study, PAL activity of ‘CDR-1’ increased post-infection and peaked at 15 dpi at level considerably higher than that of ‘Gisela 6’ (Additional file 1: Fig. S7a) reported by Liang et al. (2019). PAL is the key rate-limiting enzyme in the phenylpropanoid pathway, catalyzing phenylalanine to trans-cinnamic acid, and then synthesizing the precursors for lignin or flavonoid biosynthesis [31, 32]. For the treatment with inoculation, the results here showed that POD activity began to increase during the first 10 days post infection, whereas PPO activity increased in the last 10 days. In the study of Liang et al. (2019), POD and PPO activity of ‘Gisela 6’ both increased in the last 10 days (Additional file 1: Fig. S7b and c). Early activation of POD seemed to be a main difference in the defense response of the susceptible ‘Gisela-6’ and the resistant ‘CDR-1’.

POD enzyme is widely believed to catalyze the last enzymatic step in the biosynthesis of lignin, the dehydrogenation of the *p*-coumaryl alcohols [33]. Study in apple showed that the activities of PAL and POD significantly increased after *ɛ*-poly-L-lysine treatment with respect to activating the accumulation of phenolic compounds, flavonoids and lignin in apple fruits to form physical barriers restricting pathogen invasion [34]. PPO was known to be involved in the oxidation of polyphenols into quinines and the lignification of plant cells [35]. Some studies also suggested that phenol-oxidizing enzymes might participate in plant defense reactions [36, 37]. Unlike POD activity, in the early stage PPO activity did not seem to differ between susceptible Gisela-6 and resistant CDR-1 suggesting that PPO might not play an important role or not be involved in the defense response.

Following the infection treatment, CAT activity of ‘CDR-1’ plants increased at 15 dpi, but no such difference was evident between the treatment with inoculation and wounded control groups in ‘Gisela 6’ (Additional file 1: Fig. S7d) [2]. The LOX enzyme was known to contribute to defense responses against pathogenic microorganisms in many plant species, being a key enzyme for JA biosynthesis [38, 39]. LOX activity in the treatment with inoculation was significantly higher than that in the wounded control in the first 15 days after infection. The LOX activity in ‘CDR-1’ in treatment group was significantly higher compared to that in ‘Gisela 6’ (Additional file 1: Fig. S7e) [2], which was a main difference in the defense response of the susceptible ‘Gisela-6’ and the resistant ‘CDR-1’.

APX, MDHAR, DHAR, and GR are the four key enzymes in the AsA-GSH cycle, which is an efficient antioxidant system to eliminate reactive oxygen species (ROS). In our study, trends of all four enzymes in ‘CDR-1’ were generally similar to those in ‘Gisela 6’ [2], except that the APX enzyme activity was markedly higher in ‘CDR-1’ (Additional file 1: Fig. S7f). In photosynthetic organisms, APX is a very important reducing substrate for H_2_O_2_ detoxification. In some studies, it is indicated that APX activity is important in controlling the H_2_O_2_ concentration in intracellular signalling under some stress conditions and pathogen attack conditions [40]. The fact that APX activity was higher in CDR-1 suggested that this rootstock might have a higher capacity to detoxify H_2_O_2_.

SA plays a key role as a signal molecule in plants’ defense responses to biotic stress. Two pathways of SA biosynthesis have been proposed. One pathway is that SA is synthesized from cinnamate which is produced from phenylalanine catalyzed by the PAL enzyme. The other is from chorismate via two reactions catalyzed by isochorismate synthase (ICS) and isochorismate pyruvate lyase (IPL) [41]. Many studies have indicated the importance of high PAL activity for SA formation induced by pathogen in plants [42, 43]. However, in this study, the total SA content of the treatment group was lower than wounded control group during the first 10 days since infection. We interpret this to suggest cinnamate catalyzed by PAL enzyme was mainly used for lignin biosynthesis rather than SA biosynthesis (Additional file 1: Fig. S8), and we speculate that SA content in both groups might have been synthesized by the ICS pathway induced by the wounding*.*

JA functions critically in enabling plants to respond and defend against attacking pathogens [44, 45]. In our study, after the ‘CDR-1’ plants were infected, JA content was significantly higher during the whole period in contrast to the wounded control, with the trend of peaking at 15 dpi and then decreasing, and considerably higher than that in ‘Gisela 6’ (Additional file 1: Fig. S9) [2]. It was noteworthy that the antagonism between JA and SA might exist in defense responses of ‘CDR-1’ against crown gall disease. The JA and SA signaling pathways are necessary for plant resistance against pathogens. It is known that these pathways interact, sometimes resulting in antagonism between the pathways. And the antagonism between the JA and SA pathways was observed in the both chemical and biological assays, although this was asymmetric [46].

We discovered 40 DEGs in the treated plants by transcriptomic analysis. About the transcriptional activation of genes involved in early defense responses of plant, admittedly these studies to date have come to differing conclusions. For example, being treated with a non-oncogenic hypervirulent *Agrobacterium* strain, the expression of defense genes in *Ageratum conyzoides* cell cultures varied 24 h post-infection [47]. In the study of tobacco plants [48], just within 3–6 h after infected with different *Agrobacterium* strains, the transcription of defense genes increased, but this began to decline with the onset of T-DNA transfer. In contrast to those findings, the study did not show changes in the level of transcript within 4 h to 24 h, but these did increase at 48 h since infection in *Arabidopsis* [49]. After the *Agrobacterium* strain was inoculated at the base of wounded *Arabidopsis* stems, only very few defense genes were activated at 3 h post-infection [5]. Among the 40 DEGs we identified, five were part of the phenylpropanoid biosynthesis pathway: *Pm4CL2*, *PmCYP450*, *PmHCT1*, *PmHCT2*, and *PmCAD*.

The phenylpropanoid pathway is the main secondary metabolic pathway for the biosynthesis of lignin, phenols, and flavonoids in plants [35], and plays a vital role in the disease resistance of plants [31]. Facilitating the host cell wall, lignin acted as a physical barrier against pathogen infection [50]. Consistent with this view, our results demonstrated that the lignin-related genes (*PmPAL1*, *PmPAL2*, *Pm4CL1*, *Pm4CL2*, *PmCAD1* and *PmCAD2*) encoding key enzymes PAL, 4CL (4-coumarate: CoA ligase), CAD (cinnamyl alcohol dehydrogenase) were all upregulated at 5 dpi in pathogen-infected ‘CDR-1’ plants.

Given all the above results, we reasonably focused on the lignin biosynthetic pathway, measuring the lignin content at different stages after the infection treatment. Corroborating our prior analyses (physiological/biochemical and transcriptomic), lignin content increased significantly in the pathogen-infected plants. In a previous study, the results suggested a defense reaction of pathogens infected the resistant plants involving the formation of stress-induced lignin [51]. Lignin content of the wounded control group also increased, albeit this increase was significantly lower compared to the treatment group. This could be explained if physical wounding of the plant alone led to an elevated lignin content, as confirmed by Soltani et al. (2006) [52], whereas the induction effect of *A. tumefaciens* was more pronounced.

Biotic stresses in plant could be combated through phenylpropanoid modulation. Biosynthesis of lignin monomers occurs through the phenylpropanoid pathway. In this pathway, the enzyme 4-coumarate: CoA ligase (4CL) is important in catalyzing the formation of hydroxycinnamoyl-CoA esters. Subsequently, it is reduced to the corresponding monolignols (hydroxycinnamoyl alcohols) [52]. In phenylpropanoid pathway, 4CL is a key enzyme and its expression is altered in response to biotic stresses, clearly indicating the importance of 4CL in counteracting various biotic stresses [53]. The *4CL* gene is at the turning point of the general pathway to the branching pathway and plays an important role in the interaction between plants and their pathogens.

Ehlting et al. (1999) reported that in *Arabidopsis thaliana*, *At4CL3* was likely to participate in the biosynthetic pathway leading to flavonoid end products, whereas *At4CL1* and *At4CL2* were probably involved in lignin formation and in the production of additional phenolic compounds other than flavonoids [54]. JA and its related compounds played an important role in the rapid localized and systemic wound responses exhibited by plants [55–57]. It has been shown that in parsley, its *Pc4CL1* gene expression was activated by a JA treatment [58] and that stresses, such as wounding, excessive UV, and pathogen infection could activate *At4CL* gene expression in *Arabidopsis* [54]. In our study, the treatment effect of transient expression was significant, indicating that when tobacco leaves injected with the target gene *Pm4CL2* were infected by *A. tumefaciens*, the relative expression of *Pm4CL2* was markedly upregulated. Research has shown that the overexpression of *4CL* may enhance the disease resistance [59]. Our experimental functional verification of *Pm4CL2* in tobacco, combined with the damage degree and relative expression of transgenic lines and wild plants, together suggested higher expression level and earlier activation of *Pm4CL2* contributed to stronger resistance.

## Conclusions

In the defense response of ‘CDR-1’ to crown gall disease, POD, PAL, LOX enzymes and JA content significantly increased after infected treatment. Further combined with transcriptome analysis and transgenic verification, our results provide evidence that the crown gall resistance of transgenic tobacco is likely related to the lignin biosynthetic pathway, in which *Pm4CL2* functions crucially during the plant defense response to the pathogen *A. tumefaciens*. We speculate that *Pm4CL2* may confer ‘CDR-1’ an increased disease resistance through the accumulation of lignin which acts as a physical barrier against *A. tumefaciens* infection. This research lays foundation for breeding crown gall-resistant cultivars in cherry production.

## Methods

### Plant materials

Cherry rootstock ‘CDR-1’ seedlings were propagated by cutting in a greenhouse. The cuttings were collected at cherry experiment and demonstration station of Northwest A&F University, Zhouzhi, Xi’an, Shaanxi, China (E 108°14.803′, N 34°11.560′). With the height attaining approximately 30 cm, plants at the same developmental stage were used for the *Agrobacterium* infection treatment.

Tobacco (*Nicotiana tabacum* L., cv SR1) seeds were obtained from Breeding Biotechnologies Co., Ltd., Shaanxi, China. The seeds were sown into plastic pots filled with sterile nutrition medium and placed in an illumination incubator (Type: GXZ-300B, Southeast instruments Co., Ltd., Ningbo, China) at 22 °C under 16-h light and 8-h dark photoperiod cycle. The fully expanded leaves of four- to six-week old grown plants were used for transient expression assays.

### Experimental design and inoculation protocols

Infected plants were grown in a greenhouse; at 0, 5, 10, 15, and 20 days post-infection (dpi) the infected tissues of the plants’ root neck were harvested, washed under running water, frozen in liquid nitrogen, and stored at − 80 °C until needed for further biochemical and lignin analysis.

For biochemical analysis, root neck tissues (0.1 g) at 0, 5, 10, 15, and 20 dpi were ground in 1% (w/v) polyvinylpolypyrrolidone (PVP) using a pre-chilled mortar and pestle, and homogenized in 1.2 mL of 50 mM PBS (pH 7.8) containing 1 mM ethylenediaminetetraacetic acid (EDTA) and 0.3% Triton X-100 [60], which was enzyme extract.

For transcriptomic analysis, the root neck tissues for RNA sequencing were collected from both non-infected and infected ‘CDR-1’ plants at 5 dpi. For each group, three independent biological replications were sequenced and analyzed.

*A. tumefaciens* used in this study was isolated by our team from the crown gall tissue of infected cherry trees before [2]. The incubation of *A. tumefaciens* was performed following the method described by Liang et al. (2019) [2]. The bacteria were inoculated in a lysogeny broth (LB) and incubated at 28 °C on a shaker (160 rpm) for 16–20 h. Bacteria were harvested by centrifugation (2500×*g*) for 20 min at room temperature (RT), and resuspended in sterile distilled water to a final optical density of 0.5 at 600-nm absorbance.

To ensure inoculation and promote uniform disease development, artificial wounding was applied. A 4-cm wound was inflicted on the root neck of ‘CDR-1’ plants (Additional file 1: Fig. S10), and inoculated with 20 μL of *Agrobacterium* suspension, as described by Niemeyer et al. (2014) [9]; applying sterile water (20 μL) to the wound served as the wounded control. After inoculation, the infection site was covered with plastic wrapping (Additional file 1: Fig. S10a) that was removed 2 days later (Additional file 1: Fig. S10b). After infection, the damage degree was divided into five grades: I < 20%; 20% < II < 40%; 40% < III < 60%; 60% < IV < 80%; 80% < V < 100%.

### Field emission scanning electron microscopy (FESEM) analysis

The morphological observation was performed following the method described by Liang et al. (2019) [2]. The morphology of infected tissues was examined using FESEM at 5 dpi. Plant tissue samples were washed with distilled water and cut into sections (length ≤ 7 mm, thickness ≤ 3 mm) that were fixed in 4% glutaraldehyde for 2 h at RT or 6 h at 4 °C. Samples were rinsed with 0.1 M phosphate-buffered saline (PBS; pH 6.8), four times for 10 min each, and then dehydrated using an ethanol dilution series (30, 50, 70, 80, 90%) for 20 min at each concentration, followed by dehydration with 100% ethanol performed three times (30 min each). Ethanol was then substituted by isoamyl acetate for 20 min, and the samples were allowed to dry, after which they were gold plated and visualized under FESEM S-4800 (Hitachi Ltd., Japan).

### Biochemical analysis

The biochemical detection (including POD, SOD, CAT, PPO, PAL, LOX, APX, MDHAR, DHAR, GR activity, SA and JA content) was performed following the method described by Liang et al. (2019) [2].

The activity of all the above enzymes and the levels of phytohormone were measured at 0, 5, 10, 15, and 20 dpi, and each treatment was replicated three times. Statistical significance was determined using Student’s *t*-test by IBM SPSS Statistics v21.0.

### Determination of lignin content

Approximately 0.5 g of a sample was ground with 95% (v/v) ethyl alcohol in a mortar, and then centrifuged at 3000×*g* for 7 min. The precipitate was washed thrice with 95% (v/v) ethyl alcohol, and washed three times again with a mixed liquor of ethanol: n-hexane = 1:2 (v/v), after which it was collected and oven-dried at 70 °C. Each dried sample was dissolved in a solution of 25% (v/v) acetyl bromide and glacial acetic acid, and placed in a thermostat water bath at 70 °C for 30 min. The reaction was terminated with 0.9 mL of 2 M NaOH, followed by 5 mL of glacial acetic acid and 0.1 mL of 7.5 M hydroxylammonium chloride, with the volume filled to 10 mL with glacial acetic acid. This solution was centrifuged at 2500×*g* for 5 min, after which the supernatant was measured for its absorption value at 280 nm. Lignin content was measured at 0, 5, 10, 15, and 20 dpi, and each treatment was replicated three times. Statistical significance was determined using Student’s *t*-test by IBM SPSS Statistics v21.0.

### Transcriptomic analysis

Transcriptomic analysis was performed following the method described by Liu et al. (2018) [61]. A total amount of 3 μg RNA per tissue sample was used as input material to prepare the RNA samples. Sequencing libraries were generated using NEBNext® Ultra™ RNA Library Prep Kit for Illumina® (NEB, USA) following the manufacturer’s recommendations, with index codes added to attribute the sequences to each sample. The clustering of these index-coded samples carried out on a cBot Cluster Generation System using the TruSeq PE Cluster Kit v3-cBot-HS (Illumina). After cluster generation, the library preparations were sequenced on an Illumina HiSeq platform that generated 125-bp/150-bp paired-end reads. Raw data (raw reads) in the fastq format were first processed using an in-house perl script. In this step, clean data (i.e., clean reads) were obtained by removing from the raw data those reads containing adapters or containing poly-N, and any low-quality reads. Hence, all of the downstream analyses were based on clean data of high quality. The index of the reference genome was built, using Bowtie v2.2.3, to which paired-end clean reads were aligned using TopHat v2.0.12 software; finally, HTSeq v0.6.1 was used to count the read numbers mapped to each gene.

The resulting *P*-values were adjusted using Benjamini and Hochberg control of the false discovery rate. Genes with an adjusted *P*-value < 0.05 found by DESeq were designated as being differentially expressed, and then annotated using the *Prunus avium* database (https://www.rosaceae.org/species/prunus_avium/genome_v1.0.a1) for reference.

### Gene expression analysis by quantitative real-time PCR (qRT-PCR)

According to the manufacturer’s instructions, total RNA of root neck tissues was extracted using RNAprep Pure Plant Kit (Tiangen Biotech Co., Ltd., Beijing, China). The PrimeScript™RT Reagent Kit (Takara Biotechnology Co., Ltd., Dalian, China) was used to synthesize single-stranded cDNA. The SYBR Premix Ex *Taq* Kit (Takara Biotechnology Co., Ltd., Dalian, China) was used to perform qRT-PCR on a Life Technologies QuantStudio®5. The conditions of PCR were as follows: initial incubation at 95 °C for 30 s, followed by 40 cycles at 95 °C for 5 s and 60 °C for 30 s. The primers were designed by Primer Premier v5 software (Additional file 1: Table S2). *ACTIN* and *YC-ACTIN* served as an internal reference for normalizing gene expression in ‘CDR-1’ and tobacco plants, respectively. All qRT-PCR experiments were performed in triplicate using three biological replicates and three technical replications. Statistical significance was determined using Student’s *t*-test by IBM SPSS Statistics v21.0.

### Vector construction

The full-length coding sequence of *Pm4CL2* was amplified using a forward primer (5′-GGACTCTTGACCATGGATGATATCCATTGCCTCTAATAATTCCGT-3′) containing a *Nco*I restriction site (underlined) and a reverse primer (5′-ATTCGAGCTGGTCACCTTAGGGCAATGGGGTTGGTGTGG-3′); the latter’s *Bst*EII restriction site (underlined) was inserted into the same site behind the cauliflower mosaic virus (CaMV) 35S promoter in the pCAMBIA1301 vector. The ligated construct (pCAMBIA1301-*Pm4CL2*; Additional file 1: Fig. S11) was then transformed into *A. tumefaciens* (strain EHA105) through the freeze-thaw method [62] and used for transient expression experimentation and the stable transformation of tobacco.

### Transient expression

The transient expression experimentation was performed following the method described by Niemeyer et al. (2014) [9], with some modifications. The *A. tumefaciens* (strain EHA105) harboring the recombined T-DNA constructs was inoculated in a lysogeny broth (LB) culture—it contained rifampicin 50 μg/mL and kanamycin 50 μg/mL—and incubated at 28 °C on a shaker (160 rpm) for 16–20 h. Bacteria were harvested by centrifugation (2500×*g*) for 20 min at RT, and resuspended in an MES buffer (10 mM MES hydrate, 10 mM MgCl_2_, 100 μM acetosyringone) at RT on a shaker (100 rpm) for 2 h, to obtain a final optical density of 0.5 at 600 nm absorbance. For transient expression in *Nicotiana tabacum*, fully expanded leaves of four- to six-week old grown plants were used. The *Agrobacterium* suspension (strain EHA105) was infiltrated into the abaxial side of the leaves with a needleless 1-mL syringe; the MES buffer lacking *A. tumefaciens* served as the control. The oncogenic *A. tumefaciens* isolated from crown gall tissue was also cultured as described above. After infiltration of the *Agrobacterium* suspension (strain EHA105) for 48 h, the oncogenic *Agrobacterium* suspension was again infiltrated into the abaxial side of the leaves. Relative expression was measured at 0 h and 12 h after the treatment and replicated three times.

### Generation of transgenic tobacco plants

Transgenic tobacco plants were generated according to a leaf disc-transformation protocol [9, 62], with some modifications. The *A. tumefaciens* (strain EHA105) harboring the recombined T-DNA constructs was inoculated in a LB culture—it contained rifampicin 50 μg/mL and kanamycin 50 μg/mL—and incubated at 28 °C on a shaker (160 rpm) for 16–20 h, to obtain a final optical density of 0.6 at 600 nm absorbance. Bacteria were harvested by centrifugation (2500×*g*) for 20 min at RT, and resuspended in an MES buffer at RT on a shaker (100 rpm) for 2 h, to obtain a final optical density of 0.4 at 600 nm absorbance. Leaf discs of sterile grown *Nicotiana tabacum* cv. SR1 plants were incubated in this *Agrobacterium* solution for 5 min, blotted shortly on sterile Whatman paper, transferred to Murashige & Skoog (MS) medium (MS medium containing 1.0 μg/mL 6-benzylaminopurine, pH 5.8) and incubated at RT in the dark for 2 days. After this cocultivation, the leaf discs were transferred to shoot induction media (MS medium containing 1.0 μg/mL 6-benzylaminopurine, 300 μg/mL Timentin, 15 μg/mL Hygromycin, pH 5.8) and incubated in a 24 °C growth chamber (16 h light, 8 h dark). Developed shoots of independent calli were cut off and transferred to root inducing medium (1/2MS medium containing 0.1 μg/mL naphthalene acetic acid, 300 μg/mL Timentin, 15 μg/mL Hygromycin, pH 5.8). The putative transgenic tobacco plants selected by kanamycin (50 μg/mL) were further identified by PCR. Transgenic F2 seeds were sown into plant containers with the same medium and cultivated for 4 weeks. Once identified by PCR and RT-PCR analysis, selected plants were cultivated for 3 more weeks until virulence assays were performed.

### Virulence assay with oncogenic *A. tumefaciens*

Virulence assay on stems was performed as described before, in the section above on infection tests. For these assays, the oncogenic *Agrobacterium* suspension was prepared as described earlier in the section on preparing *Agrobacterium* inoculum. Infected plants were cultivated further in the illumination incubator and their tumor incidence and development recorded. Relative expression was measured at 0 h, 12 h, 48 h, and 60 h after treatment (replicated three times).

### Statistical analysis

The average values and standard deviations were calculated using Microsoft Excel 2016, and data were presented as means ± standard deviation (S.D.) of three replicate samples. Statistical significance was determined using Student’s *t*-test by IBM SPSS Statistics v21.0.

## Supplementary information

**Additional file 1: Table S1.** Summary of the read numbers aligned onto the *Prunus avium* reference genome. **Table S2.** Information on the primers used for the gene expression analysis. F is the forward primer and R the reverse primer; accession number of gene and amplicon size of the primer pair. **Figure S1.** Gene ontology (GO) annotation and enrichment analysis of differentially expressed genes (DEGs). The horizontal axis shows the number of genes, while the vertical axis represents enriched GO term. The “*” indicates a significantly enriched GO term. **Figure S2.** Statistics of the KEGG (Kyoto Encyclopedia of Genes and Genome) enrichment. The horizontal axis represents rich factor, while the vertical axis shows the pathway involved. **Figure S3.** The phenylpropanoid biosynthesis pathway. The genes marked in red are the differentially expressed genes (DEGs)—*Pm4CL2*, *PmCYP450*, *PmHCT1*, *PmHCT2* and *PmCAD*—in this pathway. **Figure S4.** The expression levels of *PmPAL1*, *PmPAL2*, *Pm4CL1*, *Pm4CL2*, *PmCAD1*, and *PmCAD2* in ‘CDR-1’. Bars are the mean ± standard deviation (SD) (*n* = 3). Significant treatment effects are indicated with asterisks (* *P* < 0.05, ** *P* < 0.01, *** *P* < 0.001). **Figure S5.** Generation of transgenic tobacco plants. (a) Callus induction. (b) Bud differentiation. (c) Plant regeneration of the wild type plant. (d) Plant regeneration of transgenic lines with *Pm4CL2*. **Figure S6.** The amplified fragment length of *Pm4CL2* by PCR. (a), (b) and (c) Transgenic lines. (d) Wild-type tobacco plant. **Figure S7** Effect of *A. tumefaciens* infection on the activity of defense-related enzymes in ‘Gisela 6’ at 0, 5, 10, 15, and 20 dpi (Liang et al. 2019). **Figure S8.** The salicylic acid (SA) synthesis pathway and its intersection with the lignin biosynthetic pathway. **Figure S9** Effect of *A. tumefaciens* infection on the content of jasmonic acid (JA) in ‘Gisela 6’ at 0, 5, 10, 15, and 20 dpi (Liang et al. 2019). **Figure S10.** Infection of cherry tree rootstock ‘CDR-1’ with *Agrobacterium tumefaciens.* (a) shows where a 4-cm wound was inflicted on the root neck of ‘CDR-1’ plant and the site infected with *A. tumefaciens*, which was covered with plastic film for the first 2 days post-infection. (b) Plant showing the infection site without the plastic film on it. **Figure S11.** The ligated construct pCAMBIA1301-*Pm4CL2*.

## Data Availability

All data supporting the results of this study are included in the article and the additional files. The raw sequencing data were deposited in NCBI Sequence Read Archive under the accession number PRJNA663117 (https://www.ncbi.nlm.nih.gov/sra/?term=PRJNA663117).

## Abbreviations

SA: Salicylic acid
JA: Jasmonic acid
FESEM: Field emission scanning electron microscopy
dpi: Days post-infection
SOD: Superoxide dismutase
POD: Peroxidase
CAT: Catalase
PPO: Polyphenol oxidase
PAL: Phenylalanine ammonialyase
LOX: Lipoxygenase
APX: Ascorbate peroxidase
MDHAR: Monodehydroascorbate reductase
DHAR: Dehydroascorbate reductase
GR: Glutathione reductase
DEGs: Differentially expressed genes
4CL: 4-coumarate: CoA ligase
CAD: Cinnamyl alcohol dehydrogenase
WT: Wild-type
HR: Hypersensitive response
ICS: Isochorismate synthase
IPL: Isochorismate pyruvate lyase
qRT-PCR: Quantitative real-time PCR
LB: Lysogeny broth
RT: Room temperature
