# Supplementary material for: Resistance analysis of cherry rootstock ‘CDR-1’ (Prunus mahaleb) to crown gall disease
Source: BMC Plant Biol. 2020 Nov 12;20:516. doi: 10.1186/s12870-020-02673-0 (PMC7661173; doi:10.1186/s12870-020-02673-0)
Supplement: Supplementary file 1 — Additional file 1: Table S1. Summary of the read numbers aligned onto the Prunus avium reference genome. Table S2. Information on the primers used for the gene expression analysis. F is the forward primer and R the reverse primer; accession number of gene and amplicon size of the primer pair. Figure S1. Gene ontology (GO) annotation and enrichment analysis of differentially expressed genes (DEGs). The horizontal axis shows the number of genes, while the vertical axis represents enriched GO term. The “*” indicates a significantly enriched GO term. Figure S2. Statistics of the KEGG (Kyoto Encyclopedia of Genes and Genome) enrichment. The horizontal axis represents rich factor, while the vertical axis shows the pathway involved. Figure S3. The phenylpropanoid biosynthesis pathway. The genes marked in red are the differentially expressed genes (DEGs)—Pm4CL2, PmCYP450, PmHCT1, PmHCT2 and PmCAD—in this pathway. Figure S4. The expression levels of PmPAL1, PmPAL2, Pm4CL1, Pm4CL2, PmCAD1, and PmCAD2 in ‘CDR-1’. Bars are the mean ± standard deviation (SD) (n = 3). Significant treatment effects are indicated with asterisks (* P < 0.05, ** P < 0.01, *** P < 0.001). Figure S5. Generation of transgenic tobacco plants. (a) Callus induction. (b) Bud differentiation. (c) Plant regeneration of the wild type plant. (d) Plant regeneration of transgenic lines with Pm4CL2. Figure S6. The amplified fragment length of Pm4CL2 by PCR. (a), (b) and (c) Transgenic lines. (d) Wild-type tobacco plant. Figure S7 Effect of A. tumefaciens infection on the activity of defense-related enzymes in ‘Gisela 6’ at 0, 5, 10, 15, and 20 dpi (Liang et al. 2019). Figure S8. The salicylic acid (SA) synthesis pathway and its intersection with the lignin biosynthetic pathway. Figure S9 Effect of A. tumefaciens infection on the content of jasmonic acid (JA) in ‘Gisela 6’ at 0, 5, 10, 15, and 20 dpi (Liang et al. 2019). Figure S10. Infection of cherry tree rootstock ‘CDR-1’ with Agrobacterium tumefaciens. (a) show [file 12870_2020_2673_MOESM1_ESM.doc]

**Supplementary data**

**Table S1** Summary of the read numbers aligned onto the *Prunus avium* reference genome.

| **Sample name** | **Total reads (*n*)** | **Total mapped**  **reads [*n*, (%)]** | **Mapped reads (*n*)** | |
| --- | --- | --- | --- | --- |
|  |  |  | **Multiple matches** | **Unique**  **matches** |
| CK-1 | 43331742 | 27535005 (63.54) | 1171308 | 26363697 |
| CK-2 | 64761338 | 43178904 (66.67) | 1835089 | 41343815 |
| CK-3 | 63922658 | 43304401 (67.74) | 1862525 | 41441876 |
| Treatment-1 | 59306850 | 40400779 (68.12) | 1760196 | 38640583 |
| Treatment-2 | 52607280 | 35550540 (67.58) | 1477009 | 34073531 |
| Treatment-3 | 54407750 | 37532972 (68.98) | 1571376 | 35961596 |

**Table S2** Information on the primers used for the gene expression analysis. F is the forward primer and R the reverse primer; accession number of gene and amplicon size of the primer pair.

| **Gene name** | **Accession No.** | **Primers (**5’-3’) | **Amplicon size (bp)** |
| --- | --- | --- | --- |
| *ACTIN* | EC969944 | F-CTTGCATCCCTCAGCACCTT  R-TCCTGTGGACAATGGATGGA | 82 |
| *PmPAL1* | XM_021948624 | F-GAGCTGAAGGTGCTTTTGCC  R-GGGTAAGACCTGCATTCCGT | 104 |
| *PmPAL2* | XM_021971014 | F-AGAGCTGGGAACTGCTTTGT  R-TTCAAGCAGTCCAGCAGAGG | 120 |
| *Pm4CL1* | XM_021954366 | F-CCGTGCTGTCAATGTGCTTG  R-GGGAAGCGAAGCACCAGTAT | 125 |
| *Pm4CL2* | XM_021960453 | F-GCTTGGCATTCGCAAAGGAA  R-GGTTGGTTGTAGCCAAGGGA | 121 |
| *PmCAD1* | XM_021971734 | F-GCTAAAGAGCGTTTGGGTGC  R-GAGTGCTTAGCTGACACCGT | 113 |
| *PmCAD2* | XM_021956904 | F-CTGGCGTTTTGGTCATGGTG  R-GTACCGCCTACTACGCTACC | 103 |
| *YC-ACTIN* | U60495 | F-AAGGGATGCGAGGATGGA  R-CAAGGAAATCACCGCTTTGG | 106 |


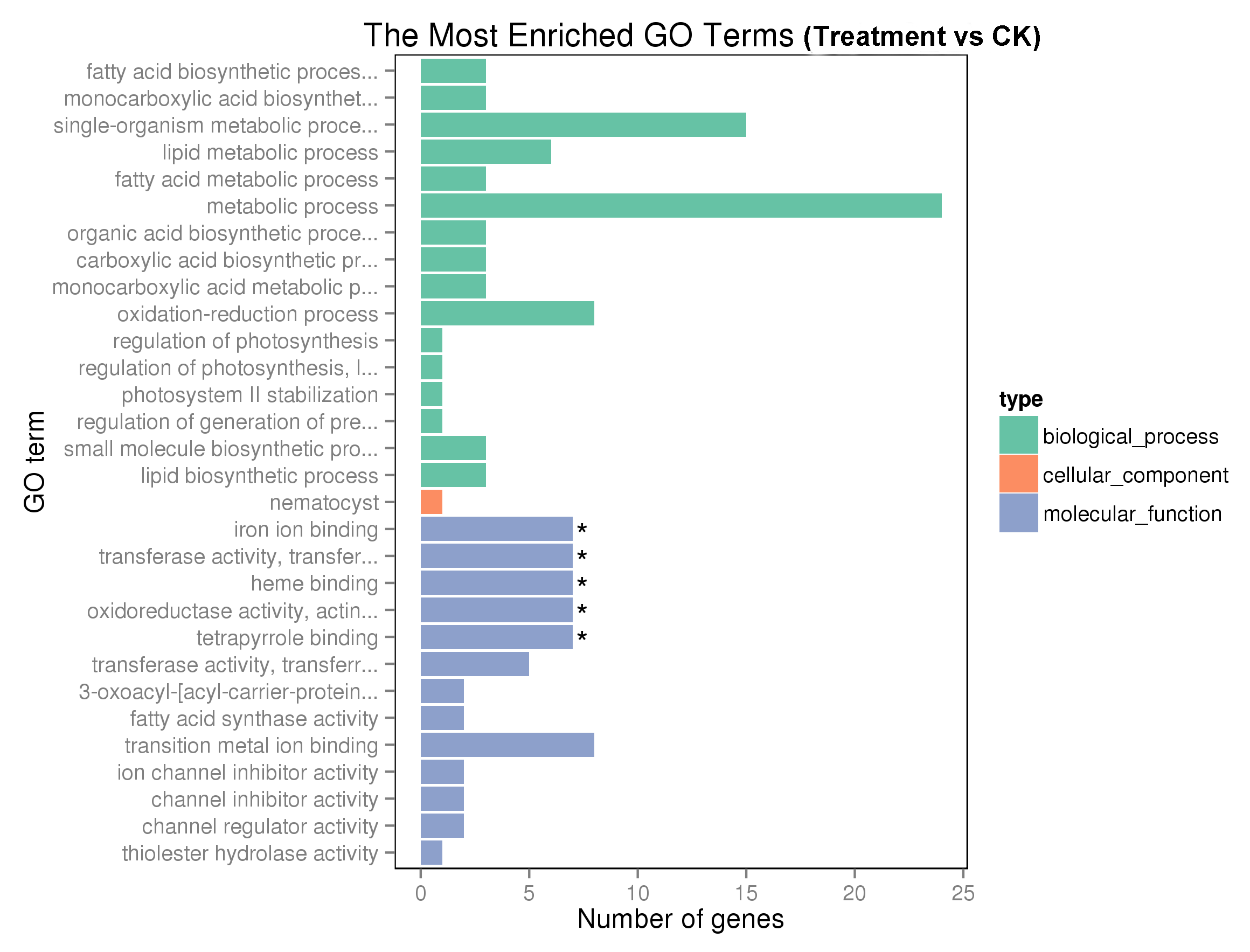


**Figure S1** Gene ontology (GO) annotation and enrichment analysis of differentially expressed genes (DEGs). The horizontal axis shows the number of genes, while the vertical axis represents enriched GO term. The “*” indicates a significantly enriched GO term.


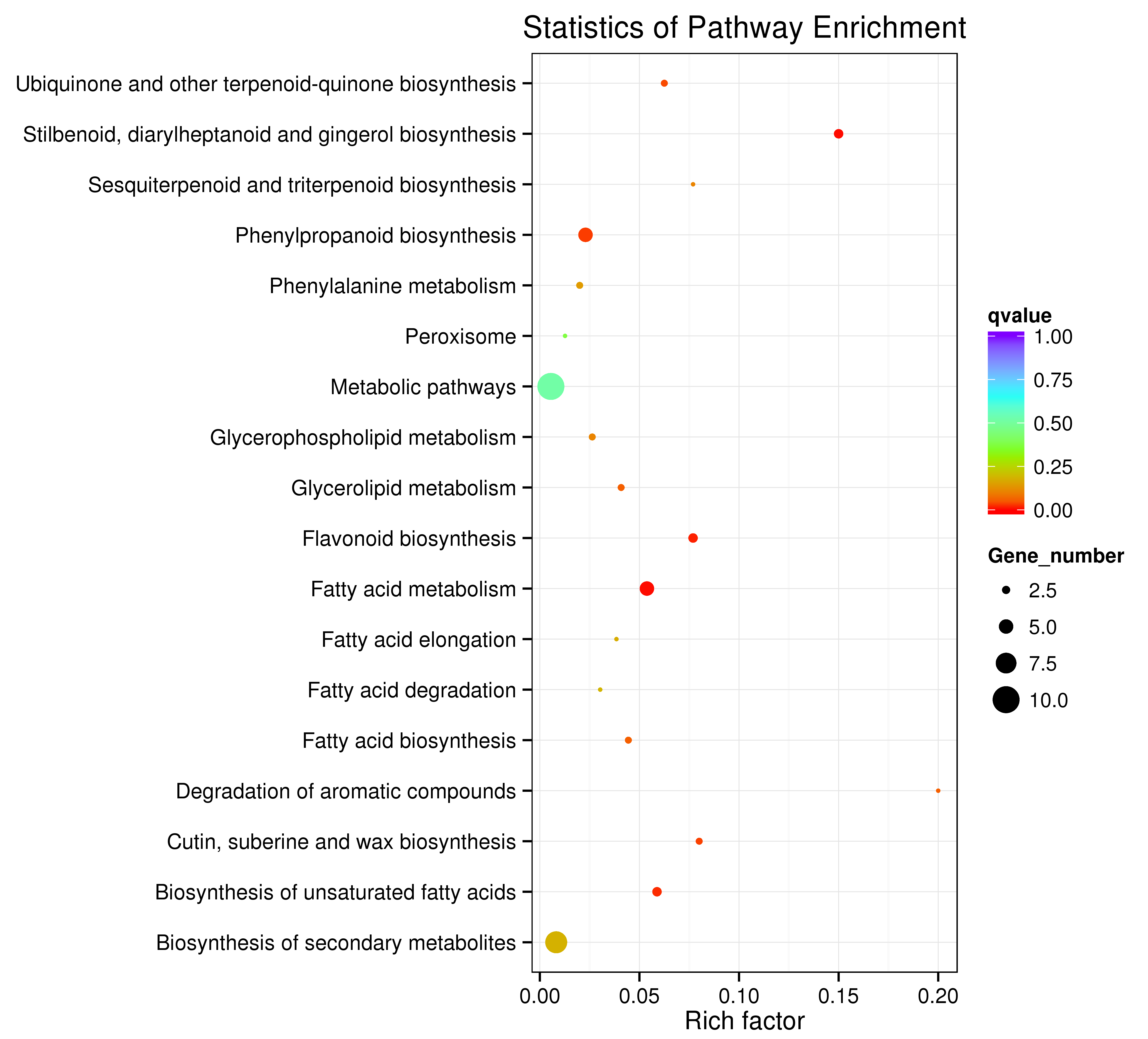


**Figure S2** Statistics of the KEGG (Kyoto Encyclopedia of Genes and Genome) enrichment. The horizontal axis represents rich factor, while the vertical axis shows the pathway involved.


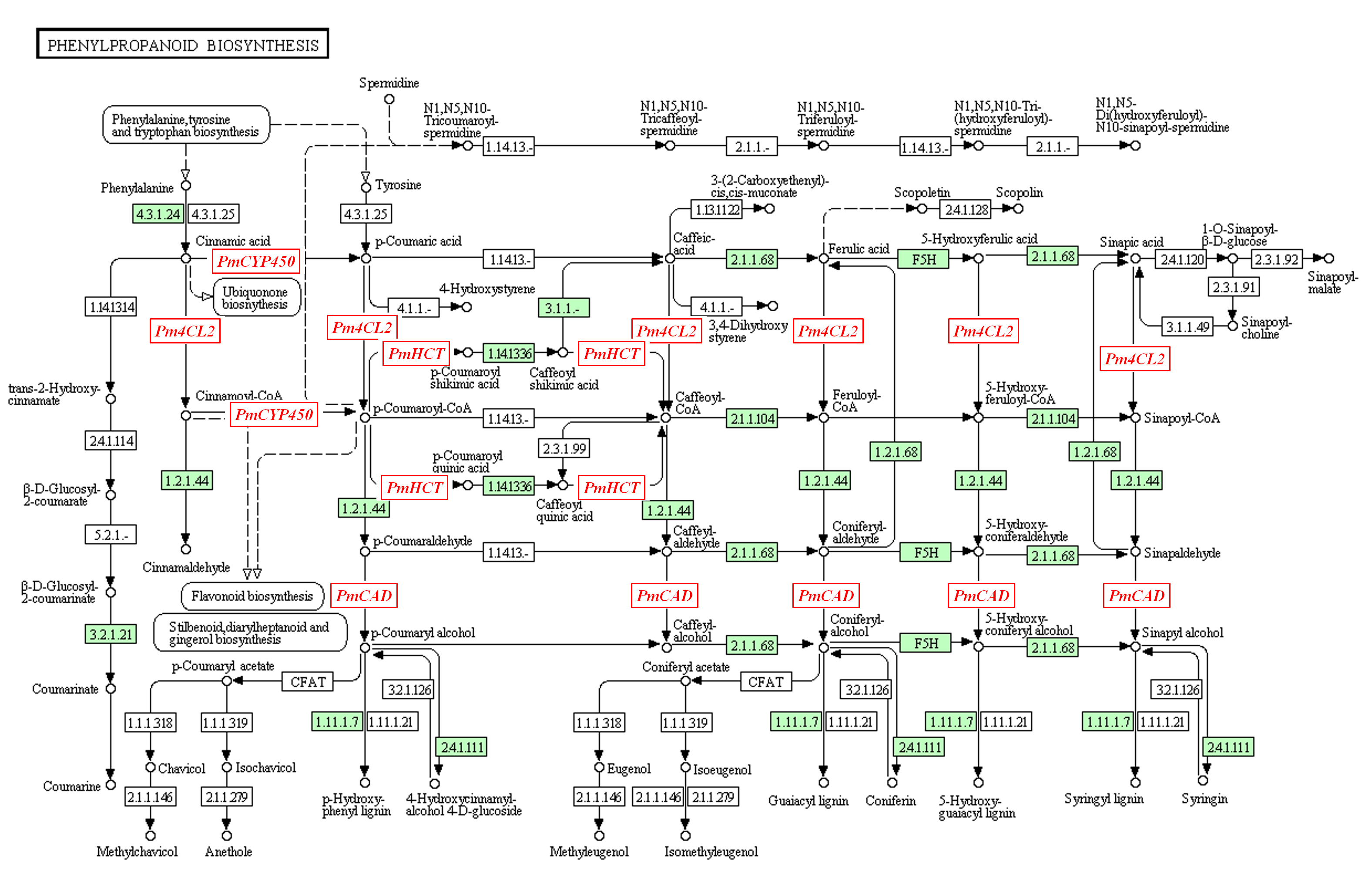


**Figure S3** The phenylpropanoid biosynthesis pathway. The genes marked in red are the differentially expressed genes (DEGs)—*Pm4CL2*, *PmCYP450*, *PmHCT1*, *PmHCT2* and *PmCAD*—in this pathway.


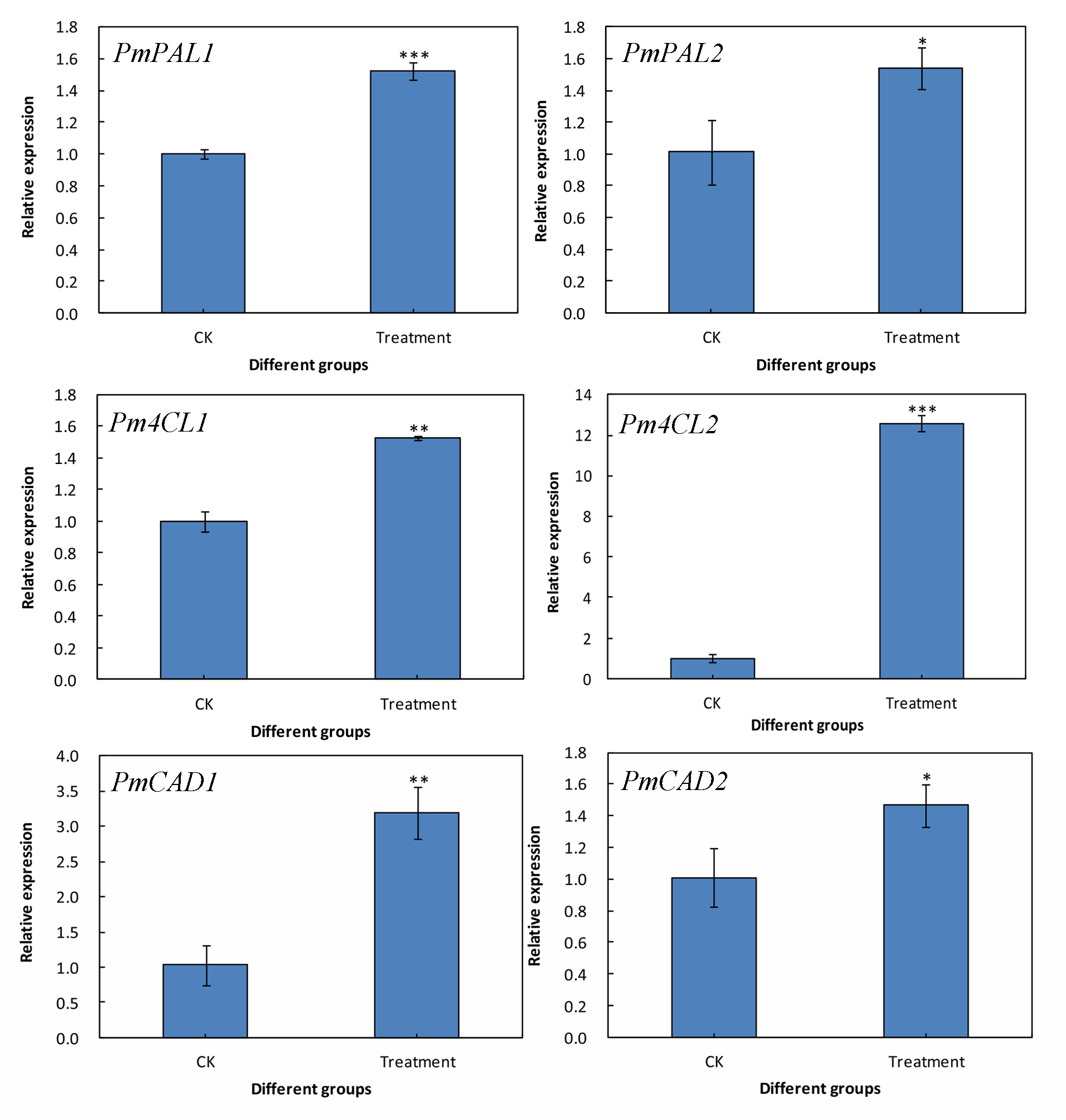


**Figure S4** The expression levels of *PmPAL1*, *PmPAL2*, *Pm4CL1*, *Pm4CL2*, *PmCAD1*, and *PmCAD2* in ‘CDR-1’. Bars are the mean ± standard deviation (SD) (*n* = 3). Significant treatment effects are indicated with asterisks (* *P* < 0.05, ** *P* < 0.01, *** *P* < 0.001).

**
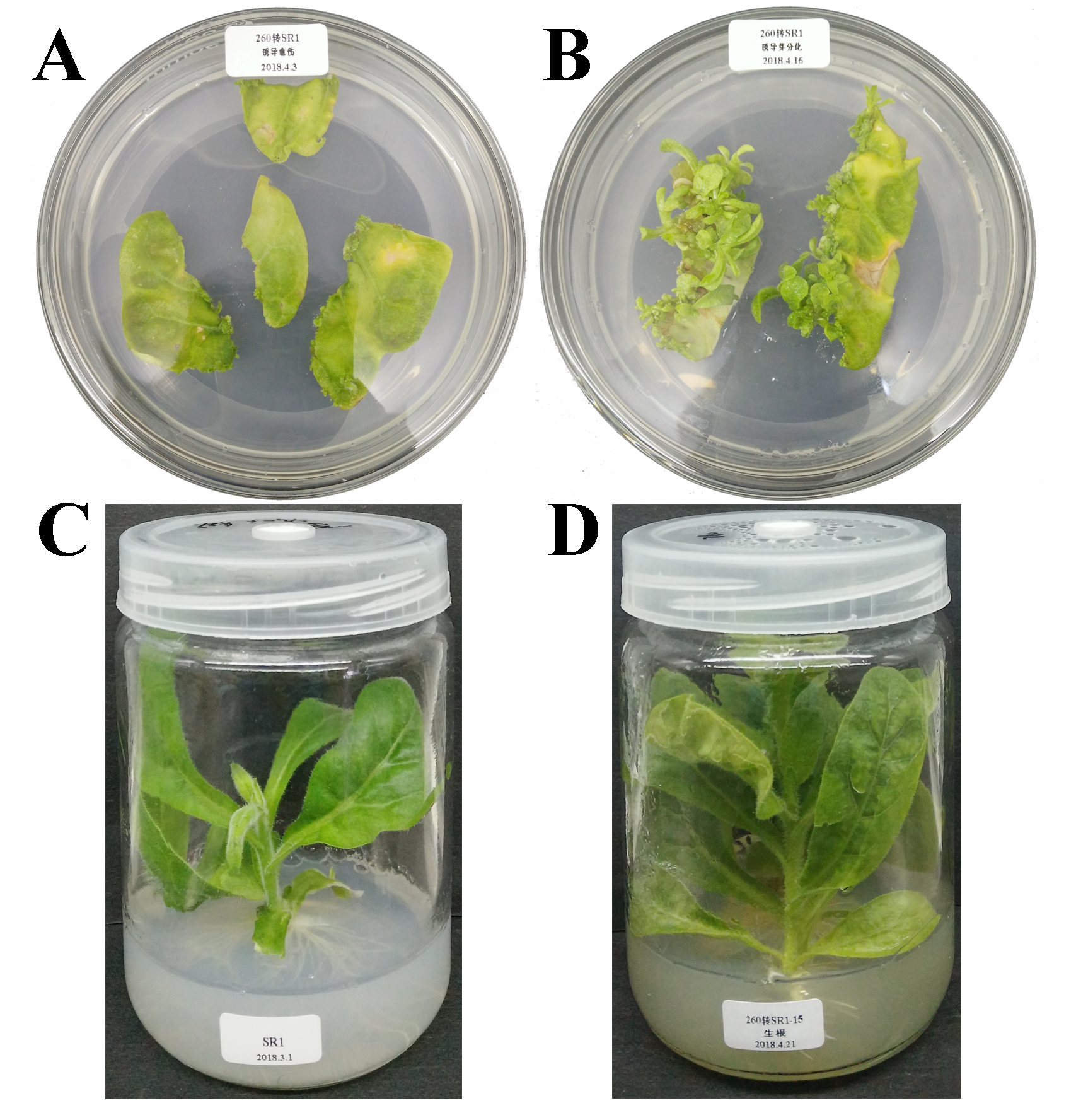
**

**Figure S5** Generation of transgenic tobacco plants. **a** Callus induction. **b** Bud differentiation. **c** Plant regeneration of the wild type plant. **d** Plant regeneration of transgenic lines with *Pm4CL2*.


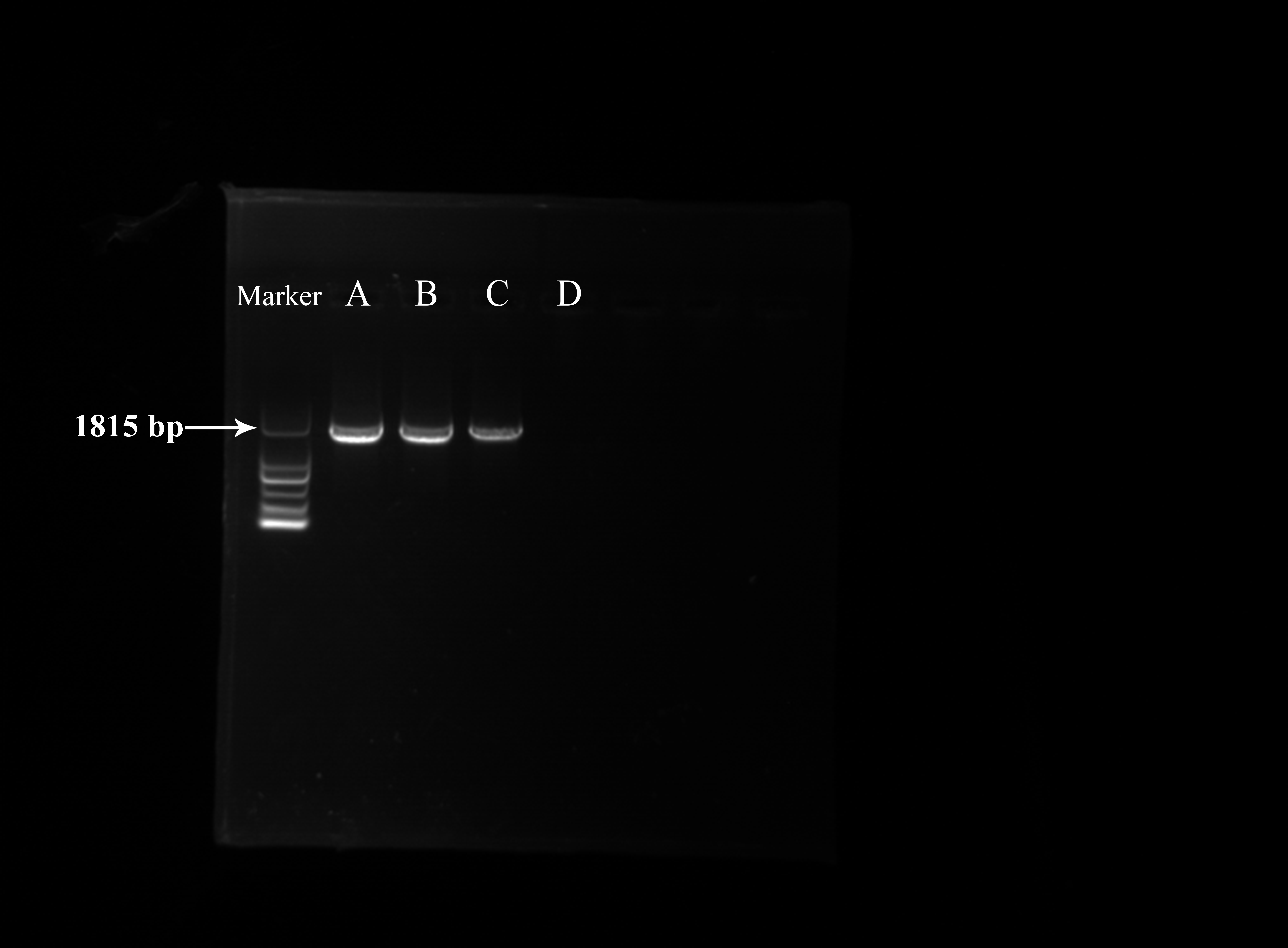


**Figure S6** The amplified fragment length of *Pm4CL2* by PCR. **a**, **b** and **c** Transgenic lines. **d** Wild-type tobacco plant.


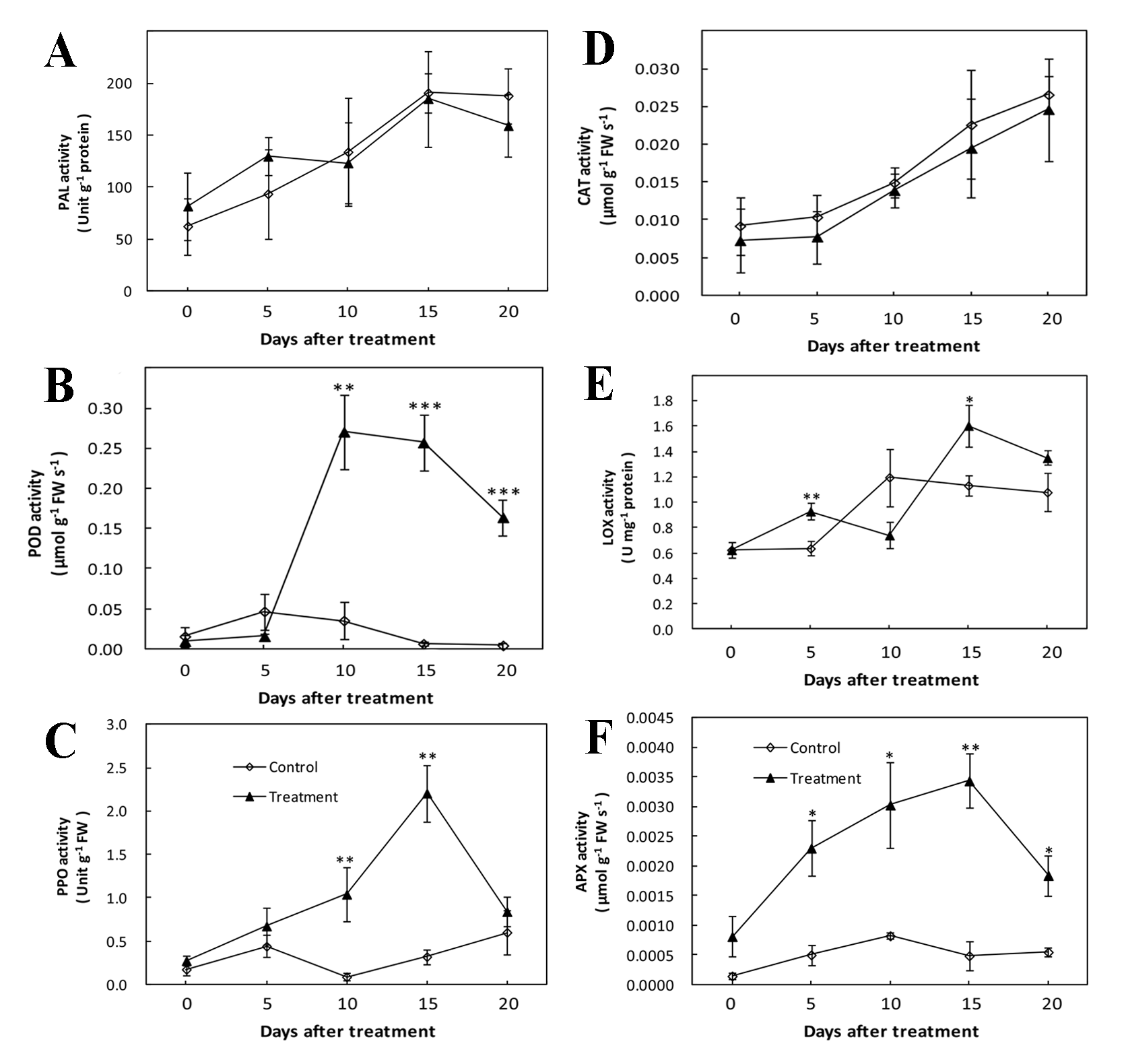


**Figure S7** Effect of *A. tumefaciens* infection on the activity of defense-related enzymes in ‘Gisela 6’ at 0, 5, 10, 15, and 20 dpi (Liang et al. 2019). **a** phenylalanine ammonialyase (PAL); **b** peroxidase (POD); **c** polyphenol oxidase (PPO); **d** catalase (CAT); **e** lipoxygenase (LOX); **f** ascorbate peroxidase (APX). Data symbols are the mean ± standard deviation (SD) (*n* = 3). Significant differences are indicated with asterisks (* *P* < 0.05, ** *P* < 0.01, *** *P* < 0.001).


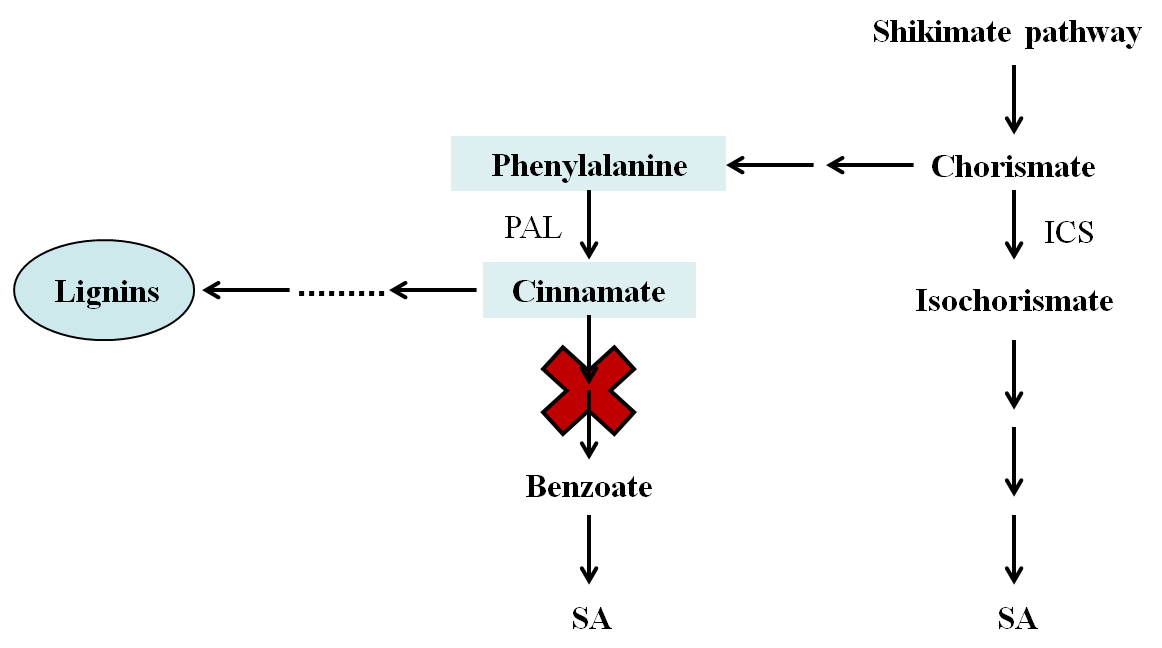


**Figure S8** The salicylic acid (SA) synthesis pathway and its intersection with the lignin synthesis pathway.


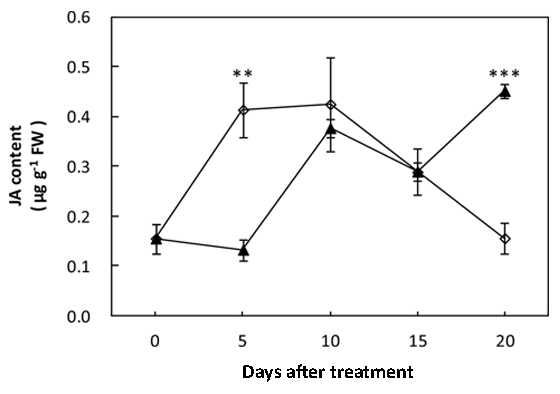


**Figure S9** Effect of *A. tumefaciens* infection on the content of jasmonic acid (JA) in ‘Gisela 6’ at 0, 5, 10, 15, and 20 dpi (Liang et al. 2019). Data represent mean ± SD (*n* = 3). Significant differences are indicated with asterisks (** *P* < 0.01, *** *P* < 0.001).


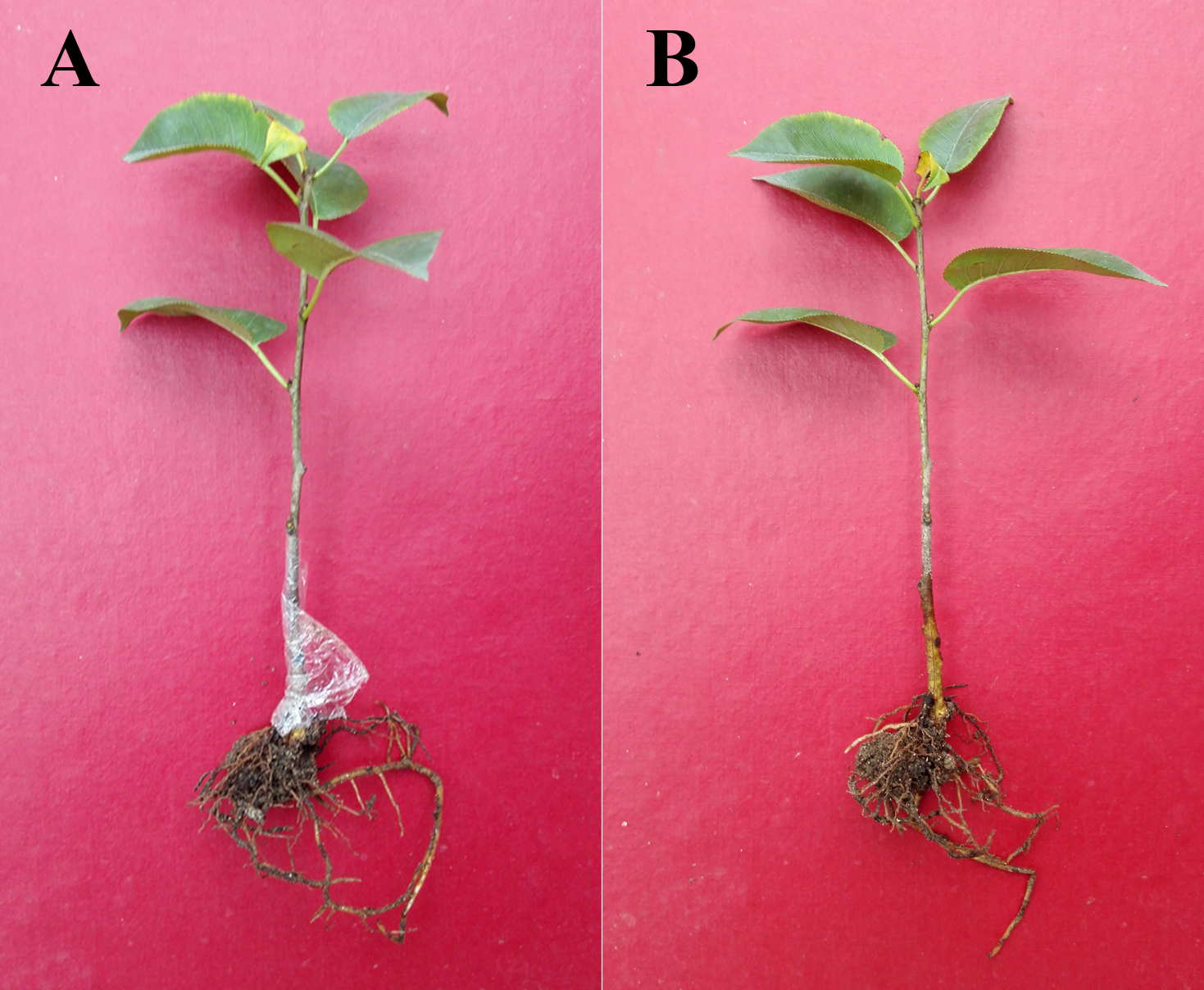


**Figure S10** Infection of cherry tree rootstock ‘CDR-1’ with *Agrobacterium tumefaciens.* **a** shows where a 4-cm wound was inflicted on the root neck of ‘CDR-1’ plant and the site infected with *A. tumefaciens*, which was covered with plastic film for the first 2 days post-infection. **b** Plant showing the infection site without the plastic film on it.


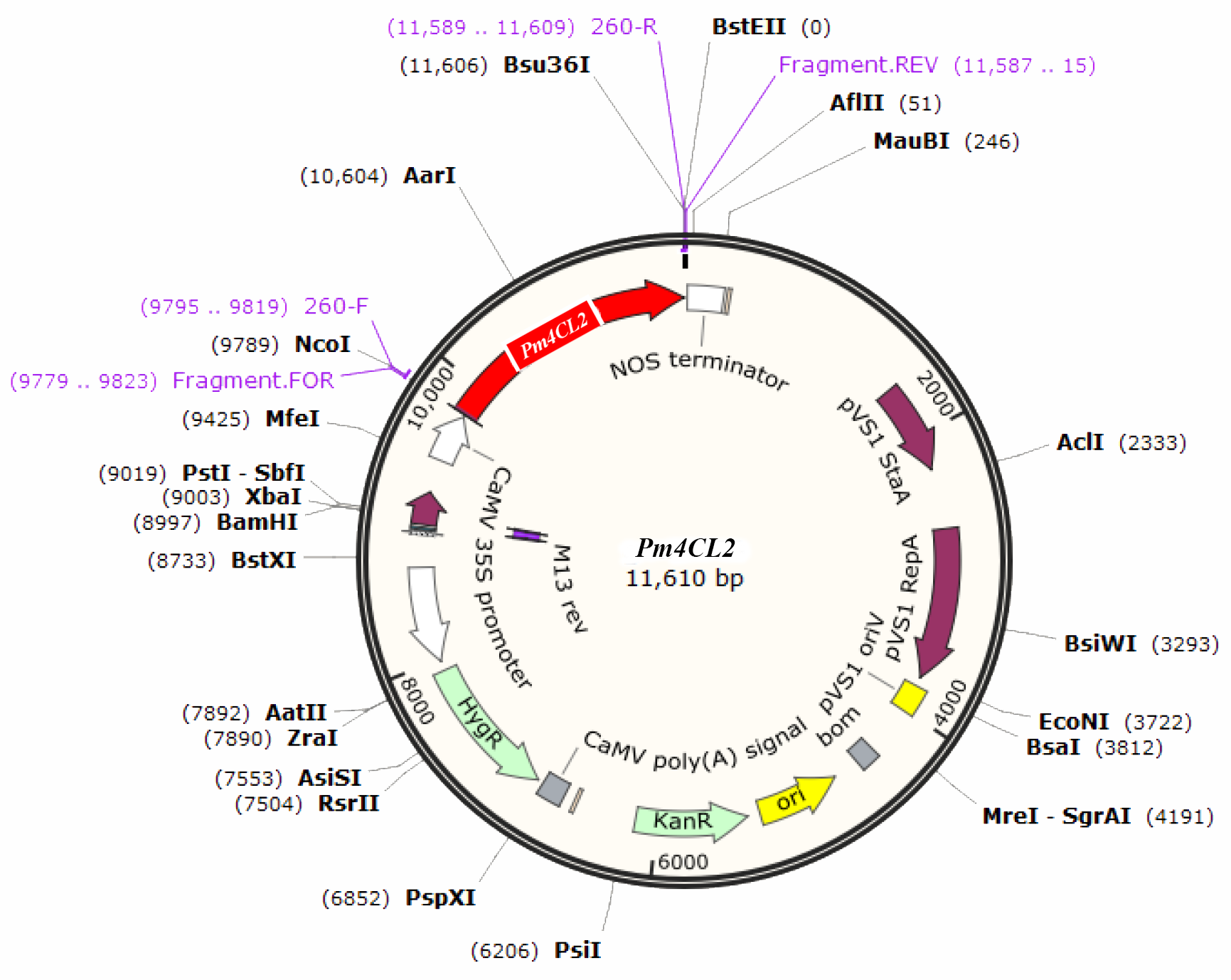


**Figure S11** The ligated construct pCAMBIA1301-*Pm4CL2*.
